# Molecular modeling studies on the interactions of 7-methoxytacrine-4-pyridinealdoxime, 4-PA, 2-PAM, and obidoxime with VX-inhibited human acetylcholinesterase: a near attack conformation approach

**DOI:** 10.1080/14756366.2019.1609953

**Published:** 2019-05-10

**Authors:** Jorge Alberto Valle da Silva, Eugenie Nepovimova, Teodorico Castro Ramalho, Kamil Kuca, Tanos Celmar Costa França

**Affiliations:** a Laboratory of Molecular Modeling Applied to the Chemical and Biological Defense (LMCBD), Department of Chemical Engineering, Military Institute of Engineering, Rio de Janeiro/RJ, Brazil;; b Faculty of Science, Department of Chemistry, University of Hradec Kralove, Hradec Kralove, Czech Republic;; c Laboratory of Molecular Modeling, Chemistry Department, Federal University of Lavras, Lavras, Brazil

**Keywords:** Acetylcholinesterase, VX, molecular modeling, NAC, 7-MEOTA-4-PA

## Abstract

7-methoxytacrine-4-pyridinealdoxime (7-MEOTA-4-PA, named hybrid 5C) is a compound formerly synthesized and evaluated *in vitro,* together with 4-pyridine aldoxime (4-PA) and commercial reactivators of acetylcholinesterase (AChE). This compound was designed with the purpose of being a prophylactic reactivator, capable of interacting with different subdomains of the active site of AChE. To investigate these interactions, theoretical results from docking were first compared with experimental data of hybrid 5C, 4-PA, and two commercial oximes, on the reactivation of human AChE (*Hss*AChE) inhibited by VX. Then, further docking studies, molecular dynamics simulations, and molecular mechanics Poisson–Boltzmann surface area calculations, were carried out to investigate reactivation performances, considering the near attack conformation (NAC) approach, prior to the nucleophilic substitution mechanism. Our results helped to elucidate the interactions of such molecules with the different subdomains of the active site of *Hss*AChE. Additionally, NAC poses of each oxime were suggested for further theoretical studies on the reactivation reaction.

## 1. Introduction

**Figure 1.**
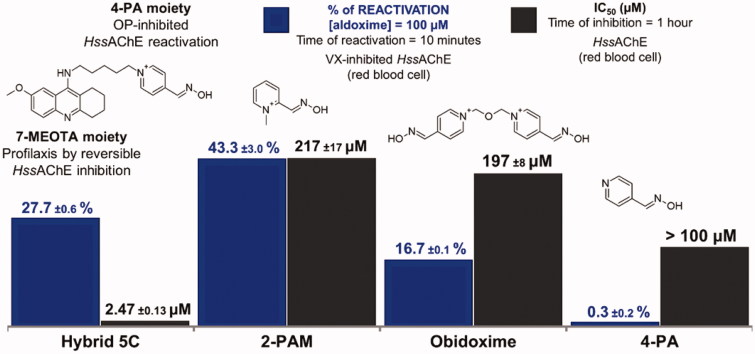
Molecular structures of some aldoximes and its experimental results for VX-inhibited *Hss*AChE, as reported by Nepovimova et al.^6^

**Figure 2.**
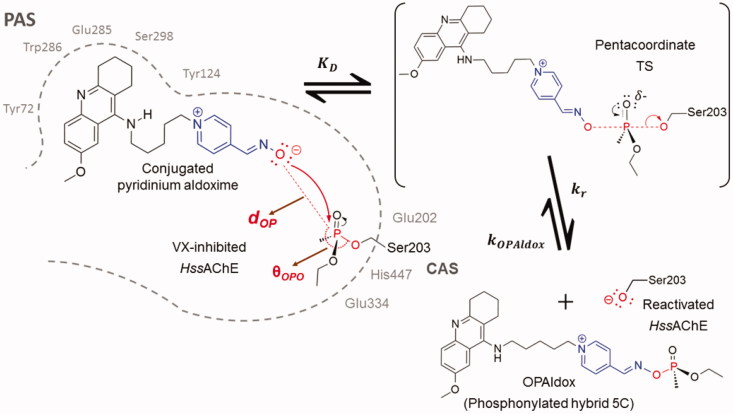
NAC approach representation. Interactions of conjugated pyridinium aldoximes with the PAS and CAS of VX-inhibited *Hss*AChE just before the reactivation reaction^1^^,^^5^^,^^7^.

2-pyridinium methyl aldoxime, known as pralidoxime or 2-PAM, was the first commercial antidote for the treatment of lethal intoxications by organophosphates (OP)^1^^,^^2^, being first administered in 1956, against parathion poisoning in Japan^3^. Since then, thousands of oximes, including the commercial bis-pyridinium aldoximes obidoxime and asoxime (HI-6), have been synthesized and evaluated. However, all these molecules were designed with the sole purpose of entering and removing the OP from the active site of the enzyme acetylcholinesterase (AChE), which is responsible for the hydrolysis of the neurotransmitter acetylcholine (ACh), and also the main molecular target of the OPs^2^^,^^4^. Recently a new approach in the design of antidotes against OPs has proposed the design of novel prophylactic enzymatic reactivators through the conjugation of the 4-pyridine aldoxime (4-PA) to a peripheral site ligand (PSL) capable of binding to the peripheral anionic site (PAS) of AChE^2^^,^^5^. Nepovimova et al.^6^ designed a novel hybrid reactivator bearing 7-methoxytacrine (7-MEOTA) as a PSL linked to 4-PA through a 5-carbon spacer (Figure 1). Ideally, this drug, named hybrid 5C by Nepovimova et al.^6^, would act in a prophylactic reversible inhibition of human AChE (*Hss*AChE) thanks to the occupancy of the 7-MEOTA moiety at the PAS, and the action of the 4-PA moiety in the catalytic anionic site (CAS; Figure 2). In case intoxication occurs, the 4-PA fragment would reactivate the phosphorylated *Hss*AChE, acting as an antidote^6^. Figure 1 summarizes former experimental results through *in vitro* tests regarding the reactivation of VX-inhibited *Hss*AChE versus *Hss*AChE inhibition by hybrid 5C^6^^,^^8^. This compound was evaluated together with the commercial reactivators 2-PAM and obidoxime, as well as 4-PA (Figure 1). According to Nepovimova et al.^6^, even though the 7-MEOTA moiety has a relatively strong capacity of enzymatic inhibition, hybrid 5C was capable of reactivating VX-inhibited *Hss*AChE better than obidoxime.

The reactivation reaction of OP-inhibited *Hss*AChE is the primary action of pyridinium aldoxime-based drugs and the most relevant mechanism for therapy against OP poisoning^1^^,^^9^. It is not a catalytic process and may proceed via the scheme described in Figure 2. After the aldoxime moiety approaches the phosphonylated Ser203 at the CAS, it may form a reversible pentacoordinate transition state (TS), followed by the displacement of the phosphonylated aldoxime (*OPAldox*) and consequent reactivation of *Hss*AChE^1^^,^^5^. The kinetic properties of this mechanism can be quantified *in vitro* by determination of the constants shown in Figure 2. The dissociation constant ($K_{D}$) is inversely proportional to the affinity of the aldoxime (ligand) to the phosphonylated *Hss*AChE (receptor), while the rate constant ($k_{r}$) indicates the aldoxime reactivity, and the rate constant ($k_{\mathrm{OPAldox}}$) is related to the re-inhibition of the reactivated *Hss*AChE by a sufficiently stable *OPAldox*^1^^,^^9^.

In this work, techniques of molecular modelling were applied within limits of docking algorithm^10^ and classical molecular dynamics (MD)^7^, considering the near attack conformation (NAC) approach^11^ (Figure 2), to analyse interactions of the complexes *Hss*AChE/VX/aldoxime studied by Nepovimova et al.^6^, and their thermodynamic contributions for reactivation of *Hss*AChE^7^. NAC is defined here as the conformation of the oxime with distance between the O of the oxime group (–C = NOH) and the P of the OP (***d*_OP_**) near to van der Waals contact, and the angle amongst the O of the oxime group, the P of the OP-Ser203 adduct, and the O of Ser203 (**θ_OPO_**), as aligned as possible. Resembling the bonds to be formed and broken in the transition state (TS) of a S_N_2 reaction^11^. Initially, poses were computed and evaluated through docking studies. Then, one pose at the NAC for each oxime was selected and submitted to rounds of molecular dynamics (MD) simulations to compute frames with atomic trajectories regarding the dynamic behaviour of ligands. Finally, molecular mechanics Poisson–Boltzmann surface area (MM-PBSA) calculations were used to computing the binding energies of the calculated frames and supporting the selection of refined NACs for future thermodynamics and kinetics studies.

## 2. Methodology

**Figure 3.**
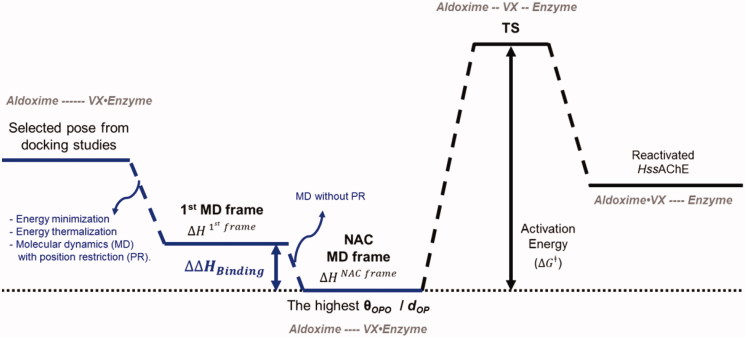
Energy profile for the whole process of *Hss*AChE reactivation. NAC: Near Attack Conformation; TS: Transition State.

The docking studies were carried out assuming that the S_N_2 mechanism^7^ takes place within the narrow gorge shaped active center of *Hss*AChE where PAS and CAS are located at the entrance and bottom, respectively^4^^,^^5^. Accordingly, computed poses were selected under the geometrical limitation of a nucleophilic attack in a NAC docked within the gorge, as illustrated in Figure 2. After, a strategic pose was selected as ligand for the MD simulations. Obidoxime and hybrid 5C were also studied as oximates (–C = NO^–^)^4^^,^^5^^,^^12^^,^^13^, once deprotonation of the –C = NOH group may happen, under physiologic conditions, in the pathway toward the NAC^5^. The blue lines in Figure 3 show the energy profile suggested as strategy employed in this work to figure out a refined NAC needed to run the further steps represented by the black lines in Figure 3. These steps correspond to deeper theoretical studies through quantum mechanics (QM) and QM associated with molecular mechanics (QM/MM) methods. These studies, besides being useful to validate the model presented in this work, will also contribute to the elucidation of the (still not fully understood) mechanism of reactivation of AChE by oximes.

### 2.1. Modeling of the oximes

Protonation states of the oximes shown in Figure 1 were estimated through the Chemicalize web-based resource supported by ChemAxon Ltd. at https://chemicalize.com/welcome14. This server predicts aqueous ionization constants (p*K*a) of organic molecules based mainly on empirically calculated partial charges and parameterized H-bonds. After that, the molecular structures were modeled with the software PC Spartan Pro 1.0.5^14^, according to the predicted protonation states, and optimized with the Merck Molecular Force Field (MMFF94)^15^. Then, their partial atomic charges were calculated through the semi-empirical quantum chemistry Austin Model 1 (AM1) method^16^ combined with the restrained electrostatic potential (RESP) model^17^.

### 2.2. Modeling of the receptor

The model of VX-inhibited *Hss*AChE was built through mutations and corrections in the crystal structure of *Mus musculus* AChE (*Mm*AChE), inhibited by tabun (GA) and complexed with HI-6, available in the Protein Data Bank (PDB) website http://www.rcsb.org/pdb^18^, under the code 3ZLV^19^. The structures of target (*Mm*AChE) and template (*Hss*AChE) present 88.60% of homological identity, with 100% of conserved residues at the active sites. Using the software Discovery Studios® Visualizer 4.5 (DS^®^ Vis 4.5)^20^, the complex *Mm*AChE/GA/HI-6 was aligned to the FASTA sequence of *Hss*AChE available at the UniProtKB data bank^21^^,^^22^, under the code UniProtKB P22303^23^. After, the complex *Mm*AChE/GA/HI-6 was manually corrected and transformed into the model *Hss*AChE/VX/HI-6, keeping all water molecules from the crystallographic structure conserved. Finally, the molecular geometry of the model was optimized through the revised version of the Chemistry at HARvard Macromolecular Mechanics (CHARMM) all-atom protein force field^24^, named CHARMM3626. The parameters of this version represent an improved model of the potential energy surface of proteins for modelling and simulation of protein dynamics in pharmacological applications. The functionally relevant conformational changes consist in newly optimized backbone torsional correction CMAP and side-chain dihedral potentials^24^^,^^25^. The first new potential was refined from experimental data on small peptides and the second one, with quantum mechanical energies from dipeptides and nuclear magnetic resonance data from unfolded proteins^24^. In addition, the new dihedral correction CMAP potential includes revised Lennard–Jones parameters for aliphatic hydrogens^26^ as well as new parameters for tryptophan^27^, being able to over stabilize helices, providing more reasonable results for the helix formed in a peptide^24^.

### 2.3. Docking studies

Prior to docking calculations, a re-docking procedure was performed to validate the protocol used. For that, the entire modelled complex *Hss*AChE/VX/HI-6 was imported to the software Molegro Virtual Docker^®^ (MVD^®^) 6.0^28^ and the crystallographic structure of HI-6 was docked over itself. The best pose from re-docking was chosen by considering the standard cartesian root-mean-square deviation (RMSD) values as well as the ligand–receptor interactions, in comparison with the crystallographic conformation of HI-6^19^. For the subsequent docking studies, the 3D structures of the ligands were also imported into MVD^®^ 6.0^28^. The cavity was detected by molecular surface with a probe size = 0.16 nm^20^ from the corrected crystallographic conformation of HI-6^19^. Using grid resolution = 0.03 nm, scaling factor = 0.5, and crossover rate = 0.9, 450 poses were generated from 15 runs, combining population sizes of 30, 50, 100, 200, and 300, with maximum iterations of 2000, 2500, and 3000. For analysis of the selected poses, interactions with the side-chains of amino acids were computed with interatomic distances up to 0.5 nm. The calculated volume and surface of the *Hss*AChE/VX cavity were, respectively, 0.326 nm^3^ and 5.491 nm^2^, and the binding site was restricted to a sphere with a radius of 1.5 nm, centered at coordinates *x* = 3.306 nm, *y* = 2.202 nm, and *z* = 1.000 nm.

Both re-docking and docking calculations were performed through the hybrid search MolDock algorithm together with the cavity prediction algorithm^29^. The docking energies were calculated in terms of MolDock Score ($E_{\mathrm{SCORE}}$), defined by Equation (1)^28^.

(1) $$E_{\mathrm{SCORE}}=E_{\mathrm{inter}}+E_{\mathrm{intra}}$$

Where $E_{\mathrm{inter}}$ is the ligand–protein interaction energy that takes into account steric approximations between atoms, based on van der Waals (vdW) interactions, a specific potential for H-bonds, and electrostatic interactions between charged atoms. $E_{\mathrm{intra}}$ is the ligand internal energy involving pairs of atoms of the ligand (except those connected by two bonds), bond torsional angles, and penalties for distances between two heavy atoms smaller than 0.20 nm^28^. A crucial parameter adopted was the percentage of selected poses at the NAC, calculated from the MolDock algorithm. Accordingly, poses were chosen, considering the distance ***d*_OP_** < 0.9 nm and the angle 140° < **θ_OPO_** < 180°, meant to provide an orbital overlapping consistent with the pentacoordinate TS shown in Figure 2^5^^,^^30–33^. Considering the random nature of the functions of the MolDock algorithm to generate initial populations and subsequent generations of poses until the final 30 per run^28^, the % of selected poses could be related to the probability of the aldoxime to be in favorable conditions of successfully reactivating the enzyme, and the highest % of selected poses might be assigned to the most promising reactivator. Therefore, the best pose of each compound was selected according to the highest value of the ratio **θ_OPO_/*d*_OP_**, presenting the nearest (lowest ***d*_OP_**) and most aligned (**θ_OPO_** closest to 180°) position.

### 2.4. MD Simulations

The best pose of each ligand selected from the docking studies was submitted to rounds of MD simulations, using the GROMACS 5.1.4 computational package^34^^,^^35^, with the OPLS/AA (Optimized Potentials for Liquid Simulations/All Atoms) force field^36^, to evaluate its dynamical behaviours, stability, and interactions inside the complex *Hss*AChE/VX^10^. Each pose was previously parameterized by the AnteChamber PYthon Parcer InterfacE (ACPYPE) algorithm^37^ to generate files of topology with parameters to be recognized by the OPLS/AA force field^36^. In addition, atomic mass and atomic partial charges of ligands were previously calculated by the software semi-empirical quantum chemistry (SQM) using the AnteChamber algorithm^38^^,^^39^. H-bond interactions were computed considering interatomic distances between heavy atoms (O and N) up to 0.4 nm, and their angle with hydrogen up to 40°.

Mulliken atomic partial charges of the ligands were calculated at AM1 level^16^ with bond charge corrections (BCC) to reproduce them as RESP^17^. Some atomic mass and OPLS/AA parameters data of such output topology files were corrected by the MKTOP algorithm^40^. Short-range vdW and electrostatic interactions were considered up to a cutoff = 1.0 nm and long-range electrostatic interactions were calculated using the Particle Mesh Ewald (PME) method^41^. The systems were subjected to energy minimizations to accommodate the water molecules and avoid overlapping of vdW radius. As before^42–44^, different algorithms with a maximum of 20,000 steps each one, were used as follows: two steepest descent minimizations, one with position restriction (STPR) up to a maximum force of 104.6 kJ mol^−1^ nm^−1^ and another without PR (ST) up to 209.2 kJ mol^−1^ nm^−1^; conjugate gradients (CG) minimization, up to 104.6 kJ mol^−1^ nm^−1^; and L-BFGS, a quasi-Newton–Raphson-based method, up to 41.84 kJ mol^−1^ nm^−1^. After, the isothermal-isochoric (NVT) and the subsequent isothermal-isobaric (NPT) ensembles were simulated to equilibrate pressure and volume of the system. Physiologic conditions were considered with *T* = 37 °C and *P* = 100 kPa, PR for the inhibited protein, leap-frog integrator, V-rescale thermostat modified from Berendsen^45^ for temperature coupling, Parrinello–Rahman barostat^46^ for pressure coupling, 2 fs of integration step, and 100 ps of simulation. Finally, the systems were subjected to 500 ps of MD simulation with PR for all atoms, except the water molecules, to ensure a balance of the solvent around the protein, and then, followed by 50 ns of MD production without restraints. Such MD simulations were run with leap-frog integrator, 2 fs of integration step, and V-rescale thermostat. Trajectories and energy data of the whole system and all its particles were obtained at every 100 ps of simulation to calculate properties such as total energy, RMSD, average number of H-bonds, and atomic distances. Output files from MD simulations were visualized with XMGRACE 5.1.25, Visual Molecular Dynamics (VMD) 1.9.3^47^, PyMOL 1.3, Molecular Graphics System 2010 Schrodinger, LLC^48^, and DS^®^ Vis 4.5^20^. The MD simulations were run in cubic boxes fulfilled by TiP4P water molecules^49^ with distance solute-wall of 1.2 nm, including periodic boundary conditions (PBC).

### 2.5. Binding energy

Integrated to the MD simulations, biomolecular interaction energies and the contributions per amino acid residue were estimated to look over key residues and their roles in the stabilization of the ligands inside the complex *Hss*AChE/VX. In that case, the MM-PBSA method^50–53^ was used through the g_mmpbsa software^52^ integrated with GROMACS 5.1.4^34^^,^^35^, to analyse the evolution of the total binding energies as a function of time. In MM-PBSA calculations, solvation properties are assessed by combining contributions to the free energy of the ligand–receptor system (${\Delta G}_{\mathrm{Binding}}$)^52–54^, expressed in Equation (2).

(2) $${\Delta G}_{\mathrm{Binding}}={{\Delta H}_{\mathrm{Binding}}-T\Delta S=\Delta E}_{\mathrm{MM}}+{\Delta G}_{\mathrm{Solvation}}-T\Delta S$$

Where each term corresponds to the variation of the respective energies from the aldoxime and the *Hss*AChE/VX complex, calculated separately, and the complex *Hss*AChE/VX/aldoxime, calculated as a whole system^52–54^. ${\Delta H}_{\mathrm{Binding}}$ and TΔS refer to the contributions of enthalpy and entropy, respectively. ${\Delta E}_{\mathrm{MM}}$ includes bonded terms (such as bond, angle, and torsion energies) as well as no bonded terms (such as vdW and electrostatic interactions)^52^.

Focused on just comparing energies of biomolecular interactions, only variations of enthalpy were considered to be binding energies in MM-PBSA calculations (${\Delta H}_{\mathrm{MM}-\mathrm{PBSA}}$)^52^, simplifying Equations (2) to (3).

(3) $${\Delta H}_{\mathrm{Binding}}={\Delta H}_{\mathrm{MM}-\mathrm{PBSA}}={\Delta E}_{\mathrm{MM}}+{\Delta G}_{\mathrm{Polar}}+{\Delta G}_{\mathrm{Nonpolar}}$$

Where solvation effects are accounted for ${\Delta G}_{\mathrm{Polar}}$ and ${\Delta G}_{\mathrm{Nonpolar}},$ using an implicit solvation model^55–57^. ${\Delta G}_{\mathrm{Polar}},$ estimated by solving the Poisson–Boltzmann equation^58^, is related to the electrostatic interaction between solute and solvent so that the polar surface is associated with the charge distribution of the solute. Binding energies were then computed individually for each residue to analyze its contribution for the biomolecular interaction, according to Equations (4)–(6).

(4) $$〈{\Delta H}_{\mathrm{Binding}}〉=\sum 〈{\Delta H}_{\mathrm{Residue}}〉$$

(5) $$〈{\Delta H}_{\mathrm{Residue}}〉=〈E_{\mathrm{MM}}〉+〈{\Delta G}_{\mathrm{Polar}}〉+〈{\Delta G}_{\mathrm{Nonpolar}}〉$$

(6) $${\Delta \Delta H}_{\mathrm{Binding}}={\Delta H}^{\mathrm{NACframe}}-{\Delta H}^{\text{first frame}}$$

Where $〈{\Delta H}_{\mathrm{Residue}}〉$ is the average of the binding energies ${\Delta H}_{\mathrm{Residue}}$ calculated for each residue through Equation (3), per new frame. Likewise, ${\Delta \Delta H}_{\mathrm{Binding}}$ represents the total binding energy to achieve the NAC during the MD simulation. ${\Delta H}^{\mathrm{NACframe}}$ is the ${\Delta H}_{\mathrm{Binding}}$ calculated by Equation (3) just for the NAC frame, selected through the highest ratio **θ_OPO_/*d*_OP_**. ${\Delta H}^{\mathrm{first}\;\mathrm{frame}}$ is the ${\Delta H}_{\mathrm{Binding}}$ also calculated through Equation (3), just for the first frame of the MD simulation, which was obtained from steps of energy minimization and thermalization of the best-evaluated pose selected by docking studies, as shown in Figure 3.

Dielectric constants of 2, 80, and 1 were considered for solute (*Hss*AChE/VX/ligand), solvent (water) and vacuum, respectively^50^^,^^52^. ${\Delta G}_{\mathrm{Nonpolar}}$ includes repulsive and attractive forces between solute (generated by cavity formation) and solvent (generated by vdW interactions)^56^^,^^57^. The Solvent Accessible Surface Area (SASA) was the only model used for ${\Delta G}_{\mathrm{Nonpolar}}$ calculation^50^^,^^52^^,^^56^^,^^57^, with surface tension simulated in 2.26778 kJ mol^−1^ nm^−2^, and probe radius = 0.14 nm. The number of grid points per area unit was set to 10 for ${\Delta G}_{\mathrm{Polar}}$ and 20 for ${\Delta G}_{\mathrm{Nonpolar}}.$ New frames were calculated by an autocorrelation function from the ones obtained through MD simulations^50^^,^^52^ so each term of Equation (3) was calculated per new frame. Output files of aldoxime and the *Hss*AChE/VX complex were visualized altogether with VMD 1.9^47^ as average frames of the new ones computed through MD.

## 3. Results and discussion

### 3.1. Modeling of the oximes and the receptor

The prevalent micro species calculated at pH = 7.4 for 4-PA, obidoxime and hybrid 5C are the ones with the oxime group protonated, while for 2-PAM, it is the oximate (Table 1). All these prevalent forms were chosen for the further molecular modelling studies. For obidoxime and hybrid 5C, however, we also decided to run the theoretical studies with the deprotonated forms, because, even knowing that these micro species are unlikely under physiological conditions, it is likely that they can be formed inside the enzyme due to proton exchange with some amino acid^5^. Accordingly, the 3D structures of each ligand in Table 1 were built through the software PC Spartan Pro 1.0.5^15^, and submitted to the docking and MD studies.

**Table 1.** Molecular structures of ligands predicted through protonation analysis.

Aldoxime	Structure	Percentage of the micro species^a^
4-PA		96.87%
2-PAM		97.65%
Obidoxime		93.45%
Hybrid 5C		99.74%
Obidoxime deprotonated^b^		6.44%
Hybrid 5C deprotonated^b^		0.21%

a Calculated through Chemicalize web-based resource (https://chemicalize.com/welcome)^59^.

b Deprotonated ligands considered to figure out better NACs^6^.

Superposition, with SPDBViewer^®^, of the main chains of the optimized model of *Hss*AChE/XV/HI-6 and the crystallographic structure of *Hss*AChE complexed with XV and HI-6 resolved by Bester et al.^60^, available in the PDB under the code 6CQW, afforded a RMSD value = 0.20 Å, with full superposition of the active site residues. This result shows that our model is basically identical to the experimental structure and validates the model for our theoretical studies.

### 3.2. Docking studies

Figure S1 of the Supporting Information shows the re-docking results and H-bond energies for the best-docked conformation of HI-6, as well as the superposition of its heavy atoms to the crystallographic conformation. The obtained RMSD = 0.16 nm was considered enough to validate the docking protocol once literature reports that a re-docking RMSD < 0.20 nm is considered acceptable^20^^,^^21^.

**Figure 4.**
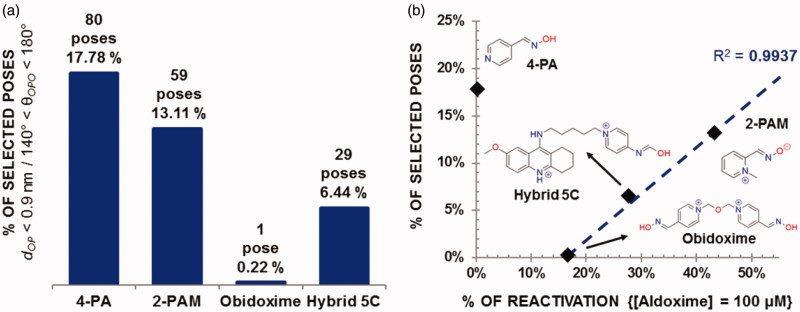
(a) Number of selected poses calculated for each oxime and (b) correlation between % of poses and % of reactivation.

The plots in Figure 4 show results for the % of selected poses according to the NAC approach^5^^,^^7^ and their correlation with the values of % of reactivation^6^^,^^8^ reported in Figure 1, while Table 2 shows the docking results for the best-selected poses of each oxime. As can be seen in Figure 4, a good correlation coefficient of *R*^2^ = 0.9937 was achieved for the % of selected poses versus % of reactivation, excluding the outlier 4-PA, the only non-cationic aldoxime under study. The alignment of the three points of the pyridinium aldoximes is in line with the correlation of the % of selected poses with the probability to be in favorable conditions for enzyme reactivation, explained at methodology. However, more points are needed to validate such theoretical correlation. Further calculations through our approach should be performed on a higher number of pyridinium oximes with experimental results (% of reactivation) available.

**Table 2.** Docking results for poses selected for MD simulations regarding the ratio **θ_OPO_/*d*_OP_**.

Ligand	$d_{\mathrm{OP}}$ (nm)	**θ_OPO_**/***d*_OP_** ± SD^a^ (nm^−1^)	$E_{\mathrm{inter}\;}\;$(kJ/mol)	$E_{\mathrm{Hbond}}\;$(kJ/mol)	H-bond interactions^b^	Hydrophobic interactions (π–π)^b^^,^^c^
4-PA^d^	0.81	185.19 ± 8.37	–328.58	–18.89	Tyr124	Tyr72, Tyr124, Trp286, Phe295, Phe297, Phe299, Phe338
2-PAM^d^	0.77	190.17 ± 6.20	–296.57	–1.49	Tyr124	Tyr72, Tyr124, Trp286, Phe295, Phe297, Phe299, Phe338, Tyr341
Obidoxime^e^	0.87	168.14	–604.94	–18.89	Tyr124, Phe297/Ser298 (Main-chain), Ser298	Tyr72, Trp86, Tyr124, Trp286, Phe295, Phe297, Phe299, Tyr337, Phe338, Tyr341
Hybrid 5C^e^	0.51	312.58 ± 44.61	–514.65	–24.86	Tyr124, Trp286	Tyr72, Tyr124, Trp286, Phe295, Phe297, Tyr337, Phe338, Tyr341
Obidoxime deprotonated^f^	0.89	159.32	–639.46	–22.74	Tyr124, Phe297/Ser298 (Main-chain), Ser298	Tyr72, Trp86, Tyr124, Trp286, Phe295, Phe297, Phe299, Tyr337, Phe338, Tyr341
Hybrid 5C deprotonated^f^	0.47	347.10 ± 3.80	–520.35	–5.68	Tyr124	Tyr72, Tyr124, Trp286, His287, Phe295, Phe297, Tyr337, Phe338, Tyr341

a Standard deviation expressed as the mean of quantity of poses selected by docking studies. Not computed for the poses of obidoxime without –C = NO^−^ moiety (only one selected pose) and obidoxime deprotonated (only four selected poses).

b Residues in bold highlight the interactions with PAS.

c Hydrophobic interactions amongst aromatic rings in parallel.

d As can be seen in Figure S3 of the Supporting Information.

e As can be seen in Figure S4 of the Supporting Information.

f Results of new docking studies to figure out better NACs using the deprotonated forms of obidoxime and hybrid 5C^5^, as can be seen in Table 1 and Figure S5 of the Supporting Information.

The unexpected result for 4-PA can be explained by comparison of the docking poses obtained for this ligand and 2-PAM shown in Figure S2 of the Supporting Information. Figure S2(a) Supporting Information shows that the 30 poses of 4-PA docked within residues Tyr72, Tyr124, and Tr286 of the PAS. The best among these poses, shown in Figure S3(a) Supporting Information, shows the N of the pyridine ring H-bonding with the side- and main-chains of Ser298. This pose was also sandwiched between Tyr124 and Trp286 through hydrophobic interactions, seeming to be trapped by those residues, in conjunction with Tyr72. Such interactions seem to hold 4-PA, avoiding its approach to phosphonylated Ser203. In comparison, Figure S2(b) Supporting Information shows 2 out of 30 poses of 2-PAM docked outside the PAS and Figure S3 (b) Supporting Information shows the best 2-PAM pose with a higher probability of leaving the PAS toward phosphonylated Ser203. These results suggest that interactions of the free electron pair of the pyridine ring in neutral 4-PA contribute to its trapping within the PAS, which may explain the poor reactivation shown in Figure 1. On the other hand, the quaternary N of the pyridinium ring of zwitterionic 2-PAM did not show any interaction strong enough to keep it inside the PAS.

Regarding the longer ligands, Figure S4(a) Supporting Information shows the best pose of obidoxime protonated with one oxime moiety pointed toward the VX-Ser203 adduct and the other moiety H-bonding with Ser298 and keeping the PSL inside the PAS. Similarly, Figure S5(a) Supporting Information shows the best pose of hybrid 5C protonated with 7-MEOTA stuck in the PAS and the oxime moiety H-bonding to Tyr124. In both cases, the PSL in the PAS seemed to create better stability for the nucleophilic attack against the VX-Ser203 adduct.

To figure out more refined NACs, with higher **θ_OPO_/*d*_OP_** ratio, new docking studies were performed on the deprotonated forms of obidoxime and hybrid 5C (Table 1). In that case, Figure S4(b) Supporting Information shows the best-evaluated pose of obidoxime deprotonated, while Figure S5(b) Supporting Information shows the best-evaluated pose of hybrid 5C deprotonated. These two poses were also selected for the MD simulations.

### 3.3. Molecular dynamics simulations

**Figure 5.**
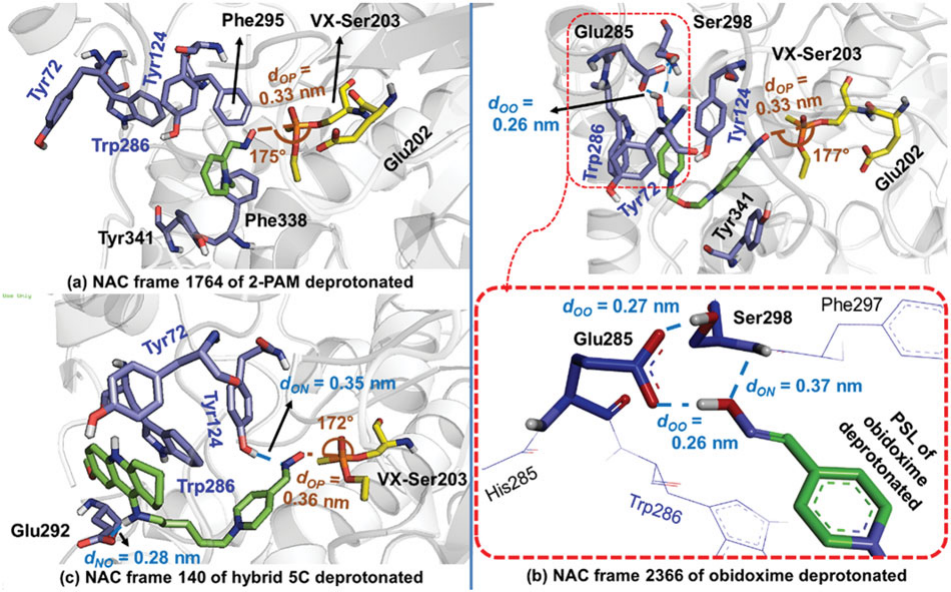
Comparisons amongst NAC frames of the ligands with the highest **θ_OPO_/*d*_OP_**.

The MD simulations were run under periodic boundary conditions (PBC) in cubic boxes of 1060 nm^3^, filled with about 32,000 TiP4P water molecules^43^. Five sodium ions were added to the systems with ligands 4-PA and 2-PAM and 7 to the other four systems. Two thousand, five hundred one frames were calculated from the trajectories computed for each system *Hss*AChE/VX/aldoxime studied. Figures S6–S8 Supporting Information show RMSD plots suggesting stability of the systems during the 50 ns of MD simulation, considering that variations never exceeded 0.35 nm for the enzyme and 0.25 nm for each aldoxime. Analysis of the dynamical behaviour of 4-PA corroborates results from docking studies, confirming that this oxime tends to be kept stuck inside the PAS due to hydrophobic π-π interactions with Tyr124 and Trp286, and H-bonds with Ser298. The ***d*_OP_** for 4-PA increased from 0.81 nm (see Figure S3(a) Supporting Information) to around 1.1 nm in the first MD frame, and stayed at this value during the rest of the MD simulation, as shown in Supporting Information Figure S9(a). Figure S10(a) Supporting Information shows the last MD frame with 4-PA still stuck within the PAS, H-bonding to Ser298. Also, Supporting Information Figure S11 shows two H-bond interactions with both side- and main-chains of Ser298 in the PAS, in agreement with the docking results. On the other hand, the dynamical behaviour of 2-PAM suggests higher stability outside the PAS, also in agreement with the docking results. For this oxime, ***d*_OP_** increased from 0.77 nm (see Supporting Information Figure S3(b)) to 0.9 nm in the first MD frame, staying at this value during the first half of simulation, then decreasing to 0.45 nm, until the end of the simulation, with approximation to the VX-Ser203 adduct, as shown in Supporting Information Figure S9(a). We believe that interaction with the main-chain of Phe295 around 20 ns, as shown in Supporting Information Figure S12, may have released 2-PAM from the PAS. The selected NAC for 2-PAM was the frame 1764, because of its highest ratio **θ_OPO_**/***d*_OP_** = 530.303 nm^−1^, with ***d*_OP_** = 0.33 nm and **θ_OPO_** = 175°, as can be seen in Figure 5(a).

The trapping of 4-PA within the PAS during the MD simulations, and the release of 2-PAM from the PAS toward the NAC, corroborated our findings from docking studies, also justifying the respective reactivation data shown in Figure 1 and the outlier point observed for 4-PA in Figure 4(b).

For obidoxime, the ***d*_OP_** first increased from 0.87 nm (Supporting Information Figure S4(a)) to around 1.0 nm in the first MD frame. Then, it decreased to around 0.4 nm, when a NAC was found within a short period around 1 ns, at frame 61, with the highest ratio **θ_OPO_**/***d*_OP_** = 331.915 nm^−1^ (with ***d*_OP_** = 0.47 nm and **θ_OPO_** = 156°), as shown in Supporting Information Figure S13. Subsequently, the ***d*_OP_** has risen to around 1.4 nm, throughout the rest of simulation, as described in Supporting Information Figure S9(a). Figure S14(a,b) Supporting Information suggest that the H of the oxime group first H-bonded to the main-chain of Thr83 from 13 ns to 18 ns of MD simulation, and then, with Glu84 from around 15 ns until the end of the simulation, reinforced by the interaction between the spacer and Tyr124. Figure S10(c) Supporting Information shows the last frame with the oxime moiety interacting with Thr83 and Glu84, presenting ***d*_OP_** = 0.62 nm. Likewise, Supporting Information Figures S14(c,d), and S15 show the H-bonds formed amongst the O of the oxime’s PSL, the main-chain of Ser298 and the two O of Glu285, during the 50 ns of MD simulation. Such interactions seem to support the formation of the NAC at frame 61, shown in Supporting Information Figure S13.

For hybrid 5C protonated, Supporting Information Figure S16 shows the H-bond with Tyr124 during the 50 ns of simulation, as also observed in the docking study (Supporting Information Figure S5(a)), suggesting that such H-bond should keep the oxime group at a NAC. However, ***d*_OP_** increased from 0.51 nm in the best-pose from docking, shown in Supporting Information Figure S5(a), to 1.15 nm away from the VX-Ser203 adduct, in the first MD frame. Then, it stayed at this value for the rest of the 50 ns of MD simulation, as shown in Supporting Information Figure S9(a). Moreover, Supporting Information Figure S17(a) shows H-bond between the oxime group and Thr83 for the first 10 ns of MD simulation, supported by interactions between Asp74 and Tyr341, as shown in Supporting Information Figure S10(d).

As discussed above, the NAC frame for obidoxime protonated was obtained only within a short period of roughly 1 ns and it was not possible to find out any NAC for hybrid 5C protonated. Such findings led us to carry out more molecular modeling studies for both obidoxime and hybrid 5C deprotonated (see Table 1), as formerly discussed in docking studies.

For obidoxime deprotonated, the ***d*_OP_** increased from 0.89 nm (Supporting Information Figure S4(b)) to roughly 1.4 nm in the first MD frame (Supporting Information Figure S9(b)). The NAC with the highest ratio **θ_OPO_**/***d*_OP_** = 536.363 nm^−1^ (***d*_OP_** = 0.33 nm and **θ_OPO_** = 177°) was found out at frame 2366, within the last 4 ns of MD simulation, as shown in Figure 5(b) and Supporting Information Figure S9(b). Figure S19(a,b) Supporting Information show the interaction between the oximate group and Tyr341 that may have helped to keep the ***d*_OP_** at 1.4 nm until 40 ns, when interaction with Tyr124 contributed to decrease ***d*_OP_** toward the VX-Ser203 adduct. Besides, Supporting Information Figures S19(c,d) and S20, show strong interactions amongst the non-protonated PSL (the nonprotonated oxime group), Glu285 and Ser298. Such interactions may have provided more stabilization to the ligand and the decreasing of ***d*_OP_** in the last 10 ns, as shown in Supporting Information Figures S9(b), S10(c), and S18(a). These interactions may also have provided stable conditions to achieve the NAC in the last 4 ns of MD simulation, as illustrated in Figure 5(b) and Supporting Information Figure S9(b).

Finally, in complete opposition to the other MD simulations, hybrid 5C deprotonated showed instability to stay in the gorge. However, some stability for this ligand was observed in the first 4 ns, as shown in Supporting Information Figure S8(b). The ***d*_OP_** first increased from 0.47 nm (Supporting Information Figure S5(b)) to around 0.5 nm in the first MD frame (Supporting Information Figure S9(b)). Then, it went to about 1.0 nm, decreased to roughly 0.45 nm and then, the NAC with the highest ratio **θ_OPO_**/***d*_OP_** = 477.778 nm^−1^ (***d*_OP_** = 0.36 nm and **θ_OPO_** = 172°) was found at frame 140, in which the ligand laid at a nucleophilic attack distance (Figure 5(c)). Accordingly, Supporting Information Figure S21 suggests H-bond interactions with Glu292 just in the first 4 ns, which seemed to be interrupted close to 11 ns. Such interactions alongside hydrophobic ones with Trp286, may have provided enough stability to finally achieving a pose at the NAC. Therefore, the NAC for hybrid 5C was found only for oximate species and with the PSL outside the PAS. In terms of dynamical behaviour, the oximate group may have provided an increasing of nucleophilicity strong enough to reach the NAC. However, the 5-carbon length of its spacer is likely too long, avoiding the appropriate fitting of the PSL within the PAS. Such difficulty may not be observed for the analogues of hybrid 5C reported by Santoni et al.^61^ inside the crystallographic structure of *Torpedo californica* AChE (*Tc*AChE) and docked inside VX-inhibited *Hss*AChE. In that case, the tetrahydroacridine moiety, stabilize inside VX-inhibited *Hss*AChE in the same way as the 7-MEOTA moiety in our study. However, the shorter spacer (4-carbon atoms) may facilitate the appropriate fitting of these molecules within the PAS to achieve the NAC.

Figure 5 shows the NAC frames of deprotonated 2-PAM, obidoxime and hybrid 5C selected with the highest ratio **θ_OPO_**/***d*_OP_**. Such selections showed a more refined conformation for nucleophilic attack with the oximate group closer to the VX-Ser203 adduct, near to the van der Waals contact (***d*_OP_** < 0.4 nm), and with more alignment amongst the O-, P-, and O- atoms. Resembling the bond to be formed in the TS (170° < **θ_OPO_** < 180°) in accordance to the definition of NAC given before^12^. The values of ***d*_OP_** < 0.4 nm observed can be correlated to the good reactivation results obtained by Nepovimova et al.^6^ shown in Figure 1, suggesting that these oximes are capable of reactivating VX-inhibited *Hss*AChE because they can adopt poses at the NAC stable enough to trigger the reactivation reaction. On the other hand, the NAC frame of protonated obidoxime, shown in Supporting Information Figure S13, also with the highest **θ_OPO_**/***d*_OP_**, present a lower refinement level than its deprotonated form shown in Figure 5(b). The same Figure 5(b) details H-bond interactions inside the PAS, amongst PSL of deprotonated obidoxime, Glu285 and Ser298, which similarly took place for both ligands, crucially holding them and impeding the exit of the respective *OPAldox*, as shown in Figure 2. Accordingly, Supporting Information Figures S19(c,d) and S20 illustrate the strength of such interactions, suggesting that such *OPAldox* could be a highly potent inhibitor, sufficiently stable to re-inhibit the reactivated *Hss*AChE, as suggested by former *in vitro* studies^1^. For hybrid 5C deprotonated, the stability observed from 4 ns in Supporting Information Figure S8(b) with the ***d*_OP_** value stuck around 0.5 nm, from 1 to 4 ns, and then continuously increasing in Supporting Information Figure S9(b), might support the exit of the respective *OPAldox* (Figure 2), after possible reactivation of the enzyme. Those chemical kinetic findings might explain the better result of reactivation for hybrid 5C than for obidoxime, despite its higher inhibition capability suggested in Figure 1.

### 3.4. MMPBSA results

**Figure 6.**
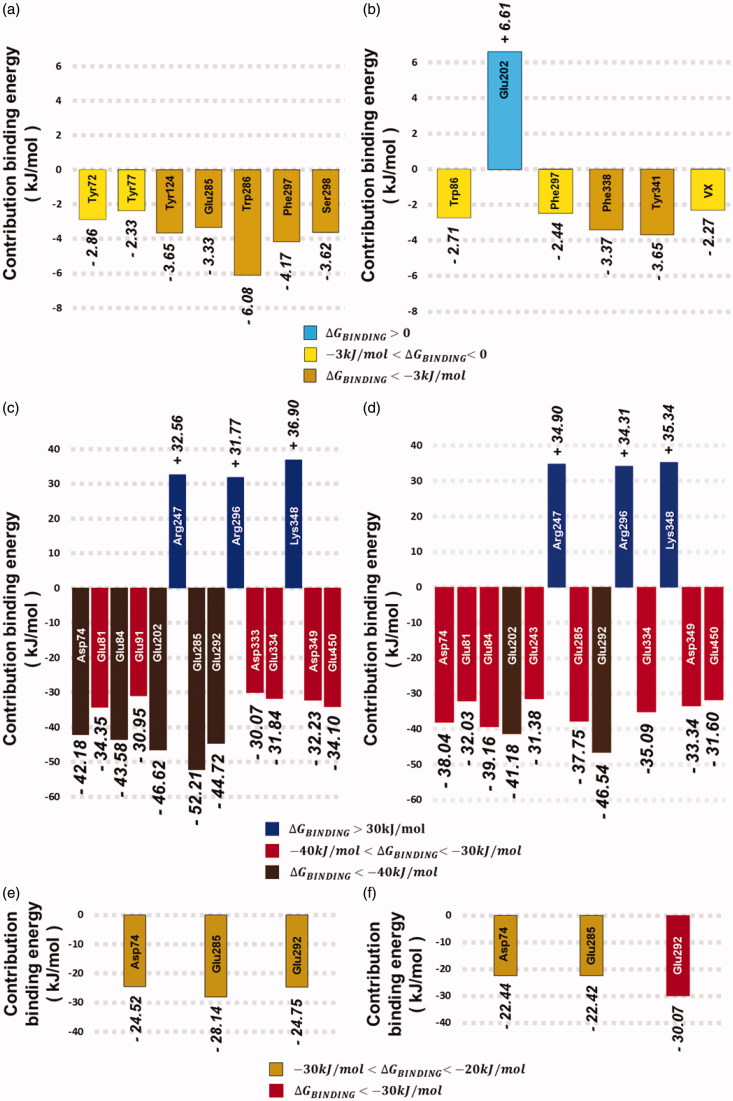
Comparisons amongst the most significant contributions $〈{\Delta H}_{\mathrm{Residue}}〉$ of key residues regarding the ligands (a) 4-PA, (b) 2-PAM, (c) obidoxime, (d) hybrid 5C, (e) obidoxime deprotonated, and (f) hybrid 5C deprotonated.

Subsequent to MD simulations, binding energies were calculated to access more details about the dynamic behavior of the oximes. Results are shown graphically in Figure 6 and illustrated in Supporting Information Figure S22 and in Supporting Information Tables S1 and S2. Analyzing Figure 6(a) and Supporting Information Figure S22(a), we can see that Trp286 and Ser298 show significant contributions for the stabilization of 4-PA within the PAS in favor of binding. Surprisingly, the contribution from Trp286 through a hydrophobic interaction seemed to be higher than the contribution from the H-bond with Ser298, calculated by MD simulation. Accordingly, Tyr72 and Tyr124 also contributed to the stabilization of 4-PA stuck in the PAS, corroborating docking and MD results. On the other hand, Figure 6(b) and Supporting Information Figure S22(b) and Table S1 suggest that the PAS residues do not contribute significantly for the stabilization of 2-PAM. Also, interactions with Phe338 and Tyr341 represent important contributions that might support the release of this oxime from the PAS, as pointed by the docking and MD results. Additionally, contributions from Phe337 and also the VX moiety, suggest a favorable path for the NAC. Otherwise, the less favorable contribution was from Glu202, given the positive value of $〈{\Delta H}_{\mathrm{Residue}}〉.$ Accordingly, electrostatic interaction between Glu202 and solvent, contributes for such instability given its high $〈{\Delta G}_{\mathrm{Polar}}〉,$ 98.26% of $〈{\Delta H}_{\mathrm{Residue}}〉,$ as shown in Supporting Information Table S1. This finding means that such positive contribution increases the $〈{\Delta H}_{\mathrm{Binding}}〉,$ according to Equation (4), increasing the instability of the system, and contributing to the ligand release from the PAS. Such findings for 2-PAM interactions are in line with studies on full-scope kinetic profile of interactions reported by Katalinić et al.^62^ where parameters of reactivation from the complexes *Mm*AChE/GA/pyridinium oximes were compared, considering the wild-type recombinant *Mm*AChE and selected mutants (Tyr337Ala, Tyr337Ala/Phe338Ala, Phe295Leu/Tyr337Ala, Tyr124Gln, Trp286Ala). In the case of the reactivation by 2-PAM, changes at PAS (Tyr124Gln and Trp286Ala) did not influence neither the affinity nor the reactivation^62^. Furthermore, the affinity for binding 2-PAM increased significantly, for mutant Tyr337Ala^62^. Accordingly, the presence of Tyr337 is indeed important for leading the oxime to serine reactivation^62^^,^^63^.

For obidoxime protonated, Figure 6(c) and Supporting Information Figure S22(c) show the strongest contribution from Glu285 which, alongside Glu202, might have supported the achievement of the NAC shown in Supporting Information Figure S13, formed at the beginning of the MD simulation. Meanwhile, along with contributions from Asp74 and Glu84, the interaction with Glu285 also might have supported the deprotonation of the oxime group through interaction with Glu84, kept at an average distance of 0.25 nm from one of the O from Glu84, between 28 ns and 45 ns, as shown in Supporting Information Figure S14(b).

To hybrid 5C protonated, Figure 6(d) and Supporting Information Figure S22(d) show a significant contribution from Asp74 which is in line with the MD simulation, seeming to attract the oxime group. Such attraction might have supported the interaction with the main-chain of Thr83 kept at a mean distance of 0.32 nm, as shown in Supporting Information Figure S17(b), likely deprotonating the oxime group, analogously to deprotonated obidoxime.

For obidoxime and hybrid 5C deprotonated, only Asp74, Glu285, and Glu292 were the most significant key residues to achieve NAC for both aldoximes. Similar to 2-PAM, their weaker contributions may provide no resistance for elimination of the *OPAldox* as shown in Figure 2. In terms of chemical kinetics, such findings corroborate that the reactivation reaction through the oximate is more effective in view of lower side-chain binding contributions. In this context, Figure 6(e) and Supporting Information Figure S22(e) show that contributions from Asp74 and Glu285, are similar to protonated obidoxime and deprotonated obidoxime, although the contribution from Glu202 has not been significant for deprotonated obidoxime. Even so, the contribution from Glu285 may have been enough to support the nucleophilic attack by the oximate as discussed before and shown in Figure 5(b). Finally, Figure 6(f) and Supporting Information Figure S22(f) show the strongest contribution from Glu292 to deprotonated hybrid 5C, which corroborates the finding that its interaction with the ligand might support the nucleophilic attack, as shown in Figure 5(c). Additionally, contribution from Trp286 also seemed to support such attack as shown in Figure 6(f), even not being significant like in Supporting Information Figure S22(f).

As a result, amongst the key residues illustrated in Figure 6 and Supporting Information Figure S22, contributions from Tyr341, Glu285, and Glu292 showed to be the most important for the chemical kinetics of the reactivation reaction through the nucleophilic attack by the oximate group of 2-PAM, obidoxime and hybrid 5C, respectively. In line with the MD simulations, such results corroborate the higher potential of the oximate for reactivation, featuring a key factor for the efficacy of the reactivation^4^^,^^5^^,^^12^^,^^13^. Such findings are also in accordance with results reported before^4^^,^^62–64^ upon docking studies of pyridinium aldoximes-based reactivators, pointing to Tyr72, Asp74, Tyr124, Ser198, Glu285, Trp286, and Tyr341, as key residues.

**Figure 7.**
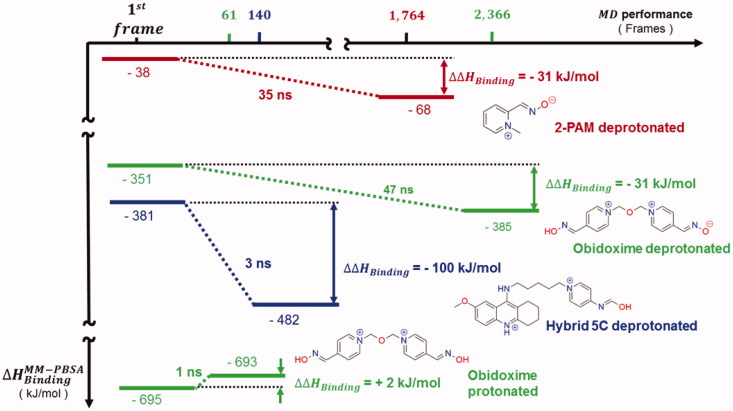
Binding energies computed for the NAC frames selected (Supporting Information Table S3).

Finally, Figure 7, drawn from Supporting Information Table S3, shows ${\Delta \Delta H}_{\mathrm{Binding}}$ values calculated according to Equation (6) for the NACs proposed, highlighting the most negative value for hybrid 5C deprotonated. Presumably, such comparison might support the thermodynamic viability of NAC for hybrid 5C deprotonated as a pathway toward the TS, as shown in Figure 2, regardless of further instability. Furthermore, the positive value for deprotonated obidoxime suggests more difficulty for the nucleophilic attack without the oximate. As a result, the NACs selected can be used in future studies of free energy of activation and reactivation dissociation with rate constants regarding affinity ligand–receptor, reactivity, and re-inhibition of reactivated *Hss*AChE by *OPAldox*^1^^,^^7,^^9^.

## 4. Conclusions

This work presented a NAC approach to theoretically investigate hybrid 5C as a reactivator for VX-inhibited *Hss*AChE, in comparison to other aldoximes. First, a percentage of 450 poses calculated through a docking protocol was selected by geometrically limiting distance and angle of nucleophilic attack against the P of the VX-Ser203 adduct. Correlations between such theoretical percentage and data of reactivation from *in vitro* tests revealed interesting findings upon structure-activity relationship of the aldoximes studied. Then, special poses from docking studies were selected and submitted to further 50 ns of MD simulations. Finally, binding energies per amino acid residue were calculated and analysed through MM-PBSA, from the 2500 frames achieved by MD. Both poses from docking and subsequent NAC frames from MD, were selected based on the ratio **θ_OPO_**/***d*_OP_**. A more refined conformation to nucleophilic attack was figured out by such criterion, especially with the oximate group closer to phosphonylated Ser203, near to van der Waals contact (***d*_OP_** < 0.4 nm), and with more alignment amongst the O-, P-, and O- atoms regarding the bonds to be broken and formed in the TS (170° < **θ_OPO_** < 180°). As a result, the NACs provided in this work for deprotonated 2-PAM, obidoxime and hybrid 5C, can be used in further structure activity relationship (SAR) studies regarding the reactivation reaction through a S_N_2 mechanism. Meanwhile, hydrophobic and H-bond interactions calculated through docking studies, were also compared with the ones analysed through MD simulations, allowing detailed analysis of the dynamical behaviour at the molecular level. Details about the role of the PAS for the docking of each ligand were also discussed. Based on binding energy calculations through MM-PBSA, contributions of such interactions with key residues were evaluated regarding the stability of the complex ligand-receptor for NAC formation. As a result, amongst the key residues discussed, contributions from Glu285, Glu292, and Tyr341 showed to be the most important to provide chemical kinetic and thermodynamic conditions to the reactivation reaction by the oximate groups of 2-PAM, deprotonated obidoxime and deprotonated hybrid 5C, respectively. The molecular modeling studies presented here with the docking protocol in conjunction with MD simulations and MM-PBSA calculations, can be a powerful set of tools to help in the elucidation of the mechanism of reactivation of AChE inhibited by OPs.

## Supplementary Material

Supplemental Material
