# Supplementary material for: Molecular modeling studies on the interactions of 7-methoxytacrine-4-pyridinealdoxime, 4-PA, 2-PAM, and obidoxime with VX-inhibited human acetylcholinesterase: a near attack conformation approach
Source: J Enzyme Inhib Med Chem. 2019 May 10;34(1):1018–29. doi: 10.1080/14756366.2019.1609953 (PMC6522925; doi:10.1080/14756366.2019.1609953)
Supplement: Supplemental Material [file IENZ_A_1609953_SM3689.pdf]

SUPPLEMENTARY INFORMATION

NAC approach for molecular modeling studies on 7-MEOTA-4-PA hybrid compound and other VX-inhibited *Hss*AChE reactivators

Jorge Alberto Valle da Silva, Eugenie Nepovimova, Teodorico Castro Ramalho
Kamil Kuca, and Tanos Celmar Costa França

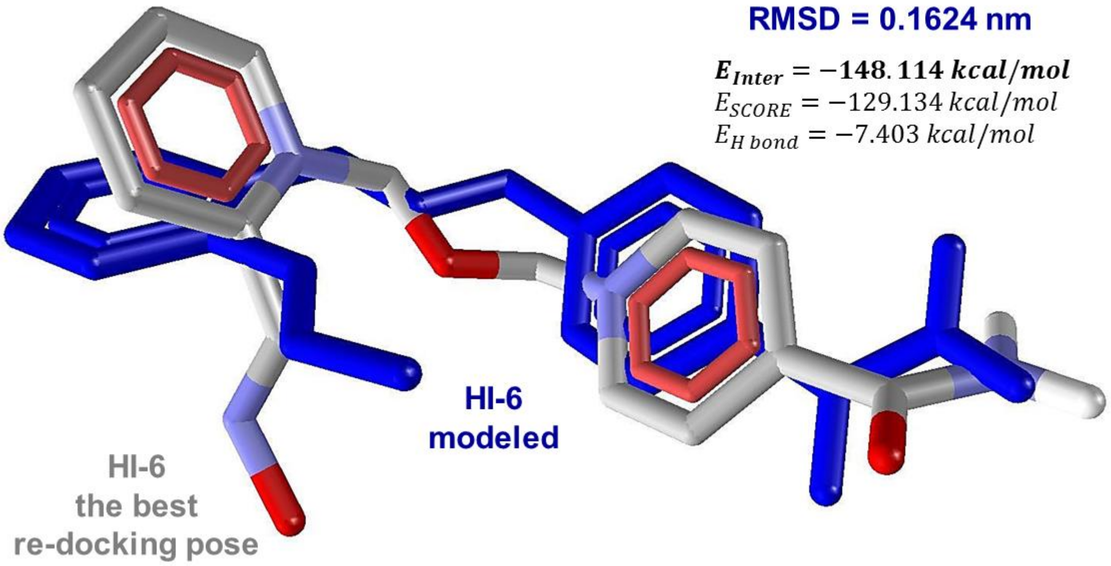

Figure S1. Re-docking evaluation of HI-6 within *Hss*AChE/VX/HI-6 complex, modeled from the *Mm*AChE/GA/HI-6 crystal complex (PDB 3ZLV).[20]

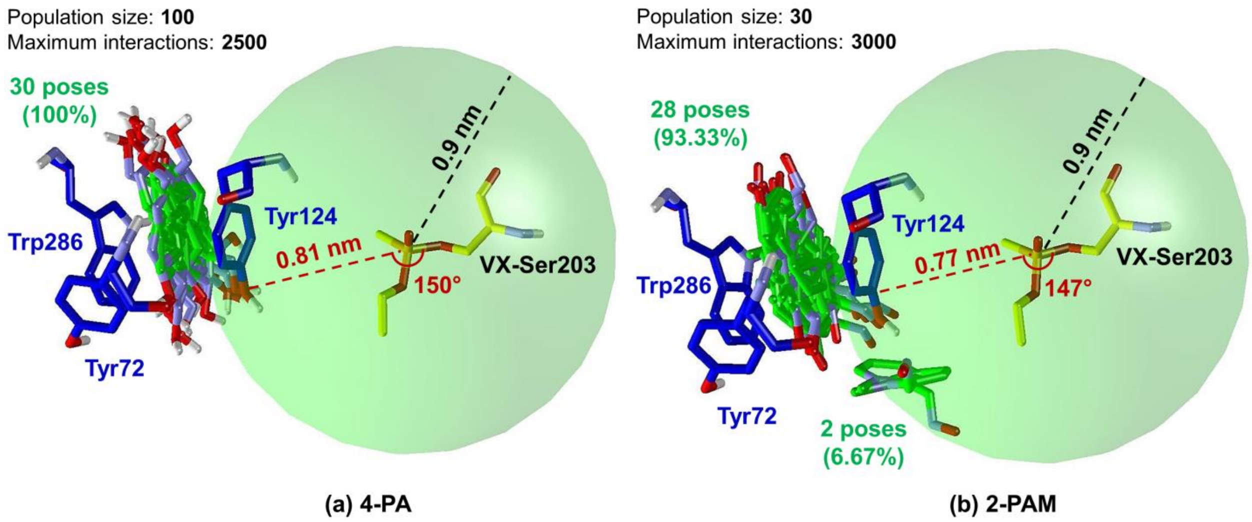

Figure S2. 30 poses computed in a run where the best-evaluated pose was selected for (a) 4-PA and (b) 2-PAM. Ligands are shown in green, VX-inhibited Ser203 of CAS in yellow and residues Tyr72, Tyr124 and Trp286 of PAS are labelled in blue. $\boldsymbol{d_{OP}}$ are rendered in red dashed lines and the search space in green spheres, with radius = 0.9 nm.

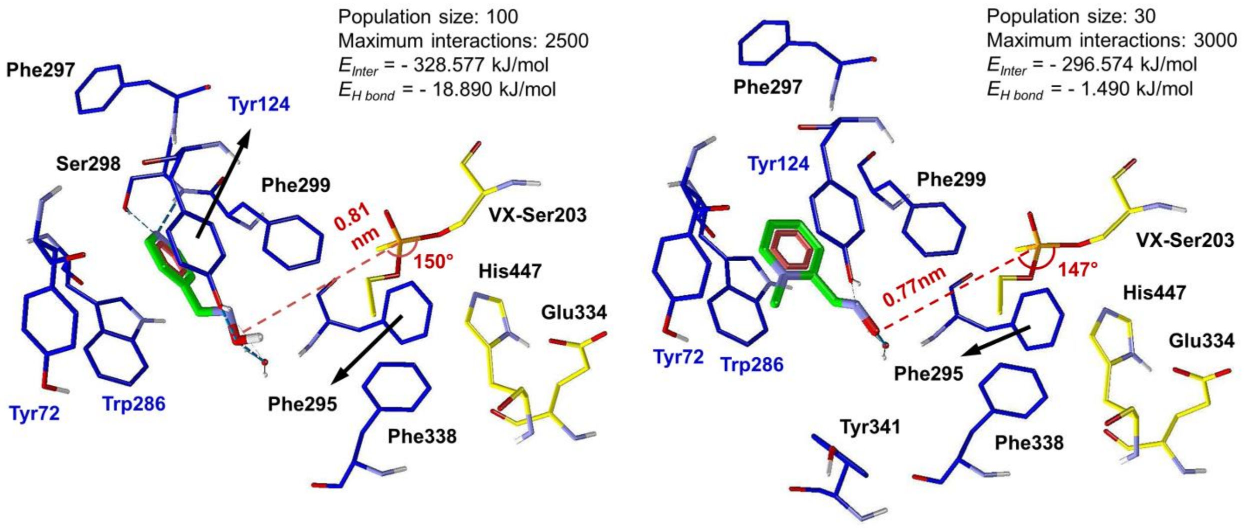

(a) The best-evaluated pose for 4-PA: with θ_{OPO} / d_{OP} = 185.19 nm^{-1}. **(b) The best-evaluated pose for 4-PA: with θ_{OPO} / d_{OP} = 190.17 nm^{-1}.**

Figure S3. Comparison between the best-evaluated poses of (a) 4-PA and (b) 2-PAM selected for MD simulations. Ligands are shown in green, residues of CAS in yellow and other residues of the cavity in blue. Residues Tyr72, Tyr124 and Trp286, belonging to the PAS, are labelled in blue. H-bonds and $\boldsymbol{d_{OP}}$ are rendered in blue and red dashed lines respectively.

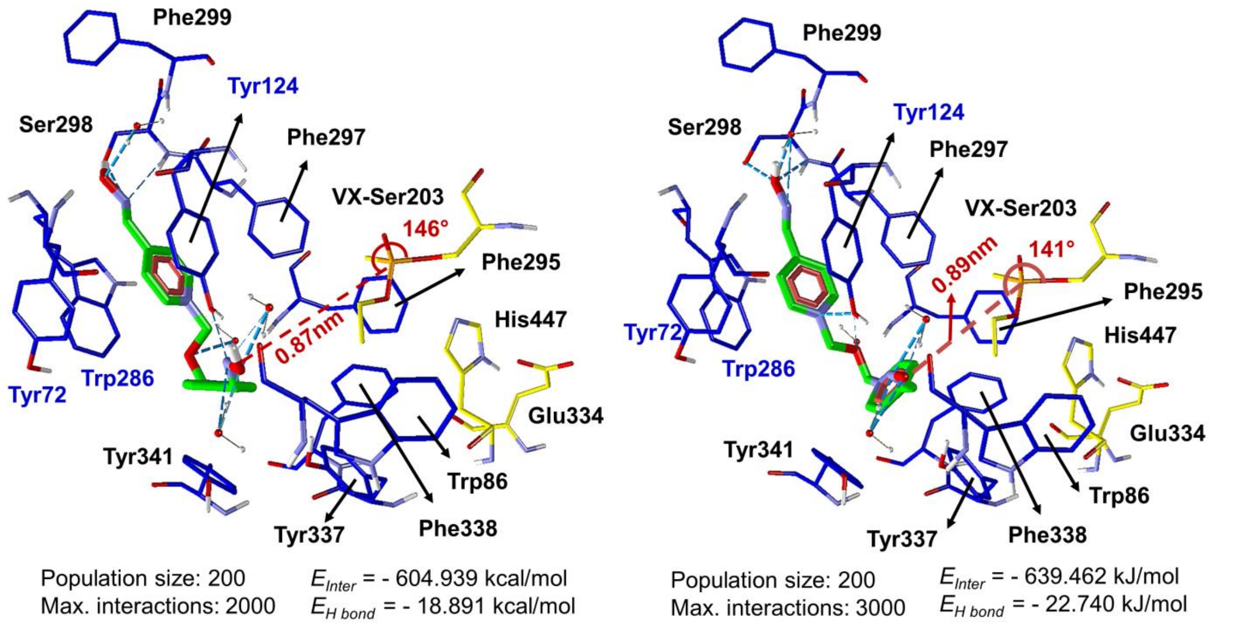

(a) The best-evaluated pose for obidoxime protonated: with θ_{OPO} / d_{OP} = 168.14 nm^{-1}. **(b) The best-evaluated pose for obidoxime deprotonated: with θ_{OPO} / d_{OP} = 159.32 nm^{-1}.**

Figure S4. Comparison between the best-evaluated poses of obidoxime (a) protonated and (b) protonated, selected for MD simulations. Ligands are shown in green, residues of CAS in yellow and other residues of the cavity in blue. Residues Tyr72, Tyr124 and Trp286, belonging to the PAS, are labelled in blue. H-bonds and $\boldsymbol{d_{OP}}$ are rendered in blue and red dashed lines respectively.

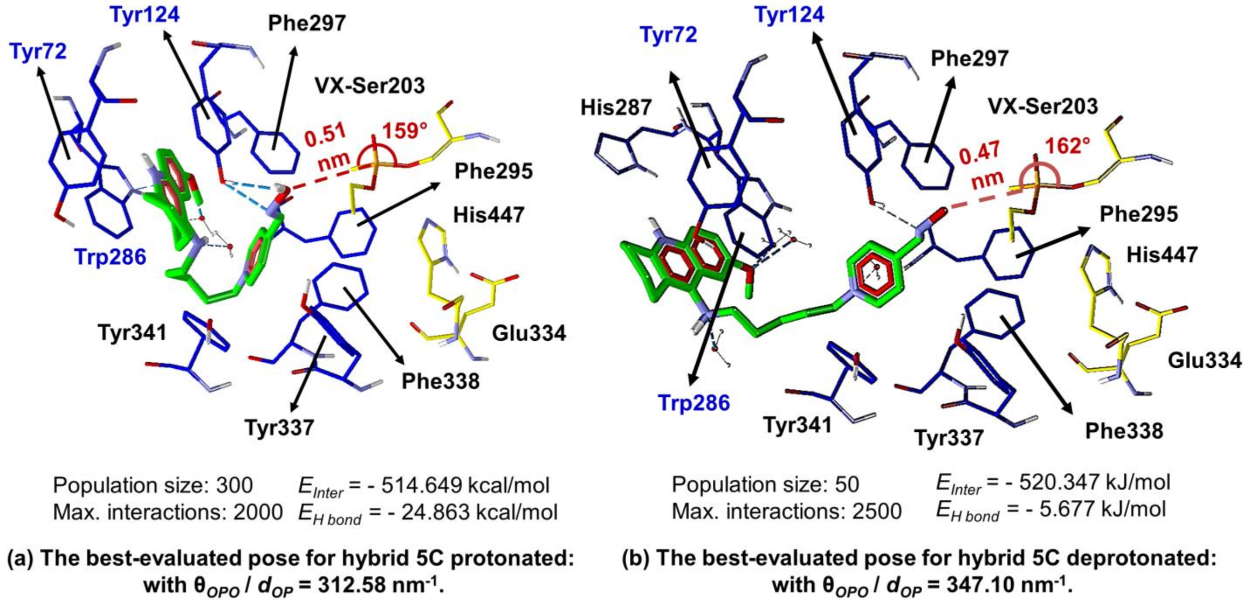

Figure S5. Comparisons amongst poses of hybrid 5C (a) protonated and (b) deprotonated. Ligands are shown in green, residues of CAS in yellow and other residues of the cavity in blue. Residues Tyr72, Tyr124 and Trp286, belonging to the PAS, are labelled in blue. H-bonds and $\boldsymbol{d_{OP}}$ are rendered in blue and red dashed lines respectively.

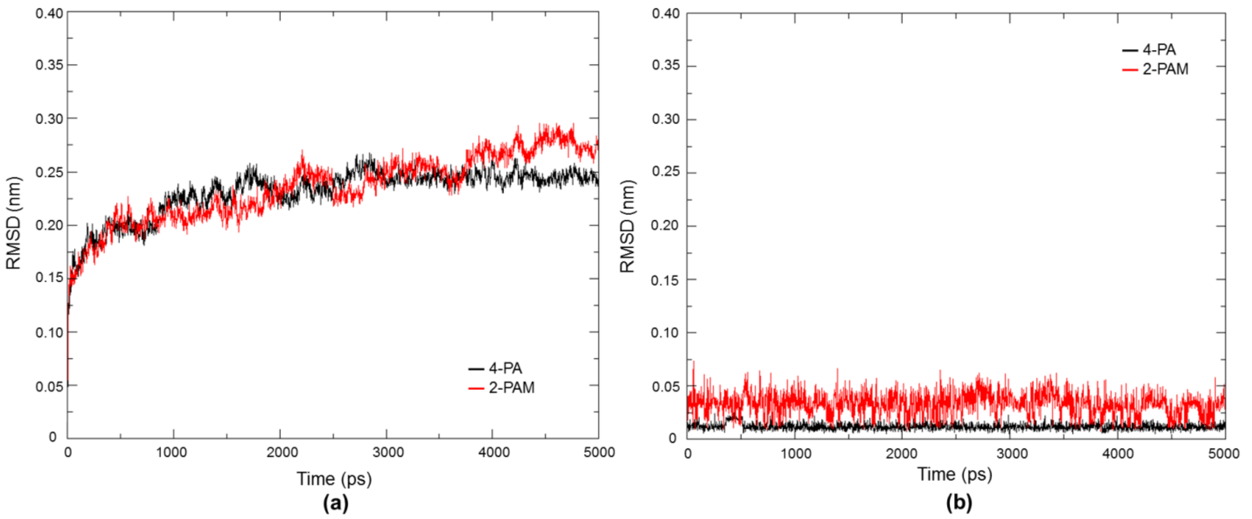

Figure S6. Comparisons between RMSD plots of 4-PA and 2-PAM throughout 50 ns of MD simulation for: (a) main-chain of both *Hss*AChE/VX complex; and (b) both ligands.

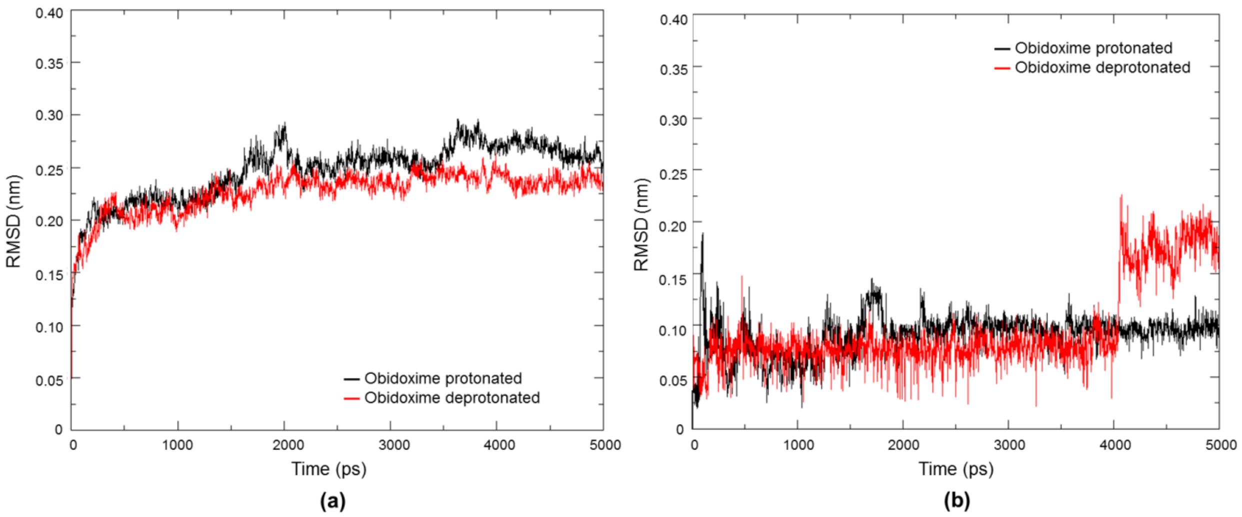

Figure S7. Comaprisons between RMSD plots of obidoxime protonated and deprotonated throughout 50 ns of MD simulation for: (a) main-chain of both *Hss*AChE/VX complex; and (b) both ligands.

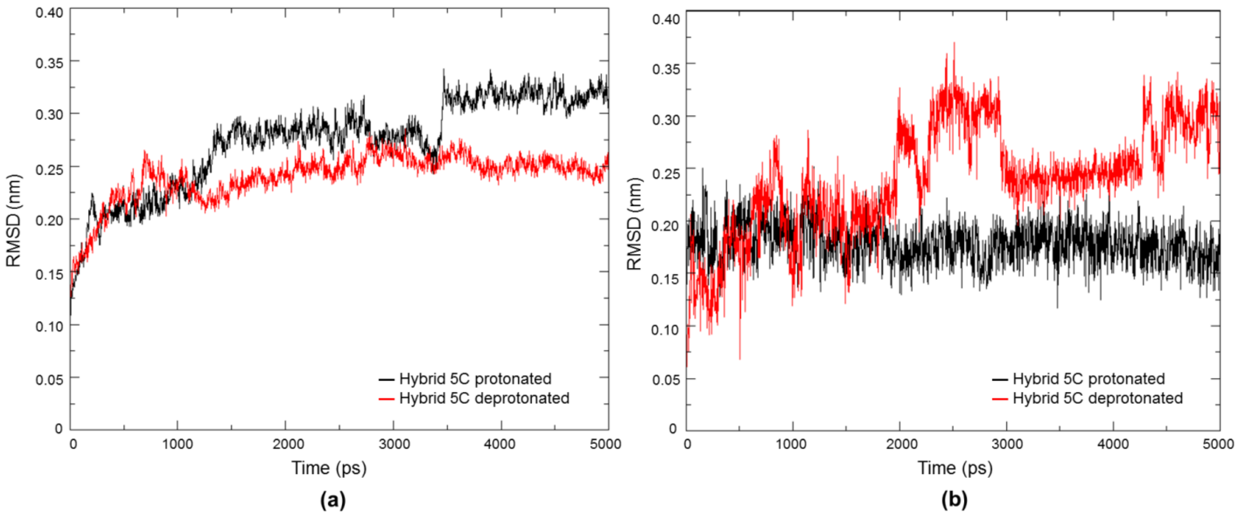

Figure S8. Comaprisons between RMSD plots of hybrid 5C protonated and deprotonated throughout 50 ns of MD simulation for: (a) main-chain of both *Hss*AChE/VX complex; and (b) both ligands.

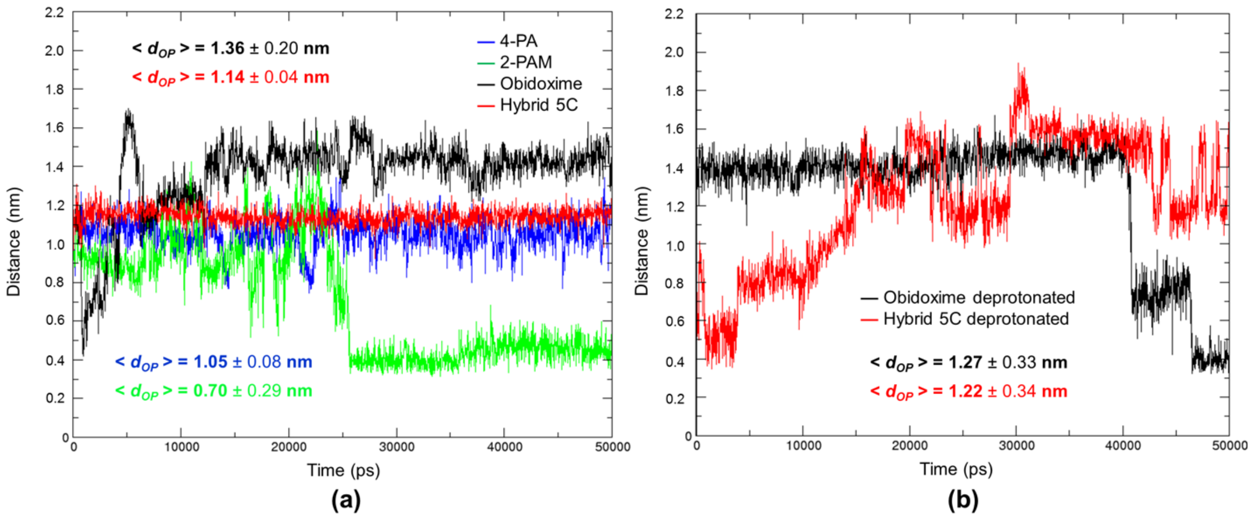

Figure S9. Comparisons amongst $\boldsymbol{d_{OP}}$ values throughout 50 ns MD simulation for each ligand. "⟨ ⟩" represents the average $\boldsymbol{d_{OP}}$ values.

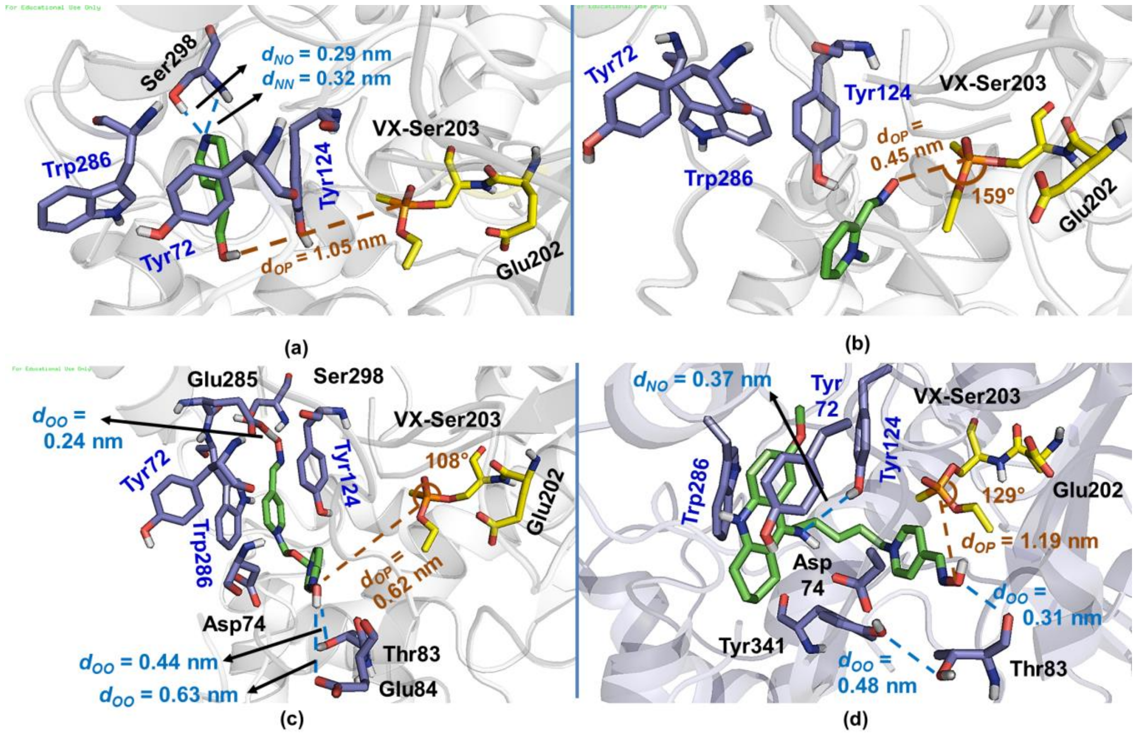

Figure S10. Comparisons amongst l[st] frames of (a) 4-PA, (b) 2-PAM, (c) obidoxime and (d) hybrid 5C. Ligands are shown in green, residues of CAS in yellow and others in blue. H-bonds and $\boldsymbol{d_{OP}}$ are shown in blue and brown dashed lines respectively.

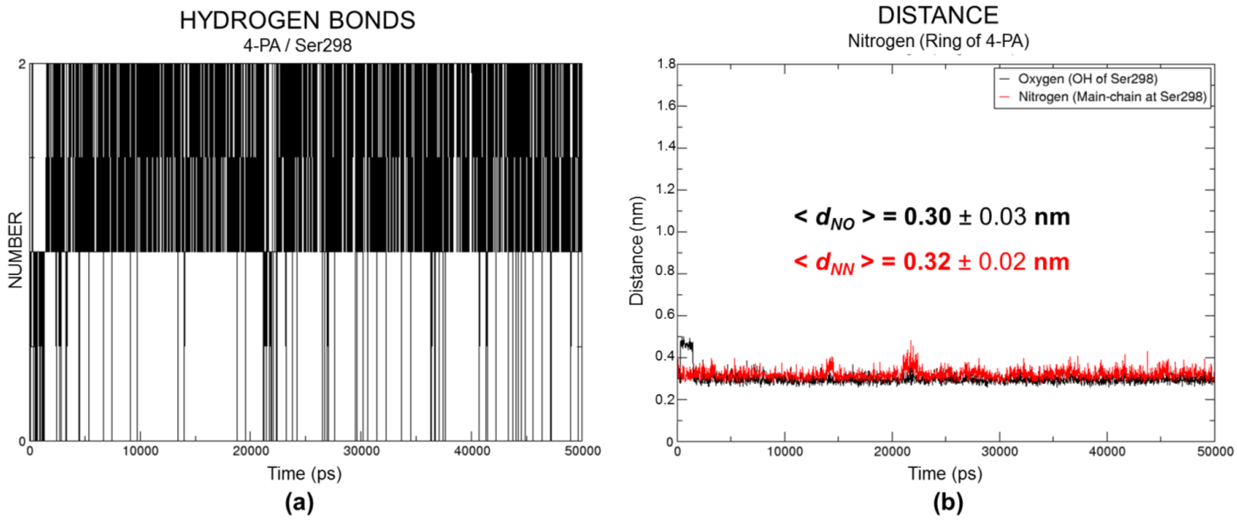

Figure S11. Plots of H-bonds between 4-PA and atoms of Ser298 (a) throughout 50 ns of MD simulation. (b) Variation of distances *d* of the H-bonds formed. "⟨ ⟩" represents the mean of *d* values.

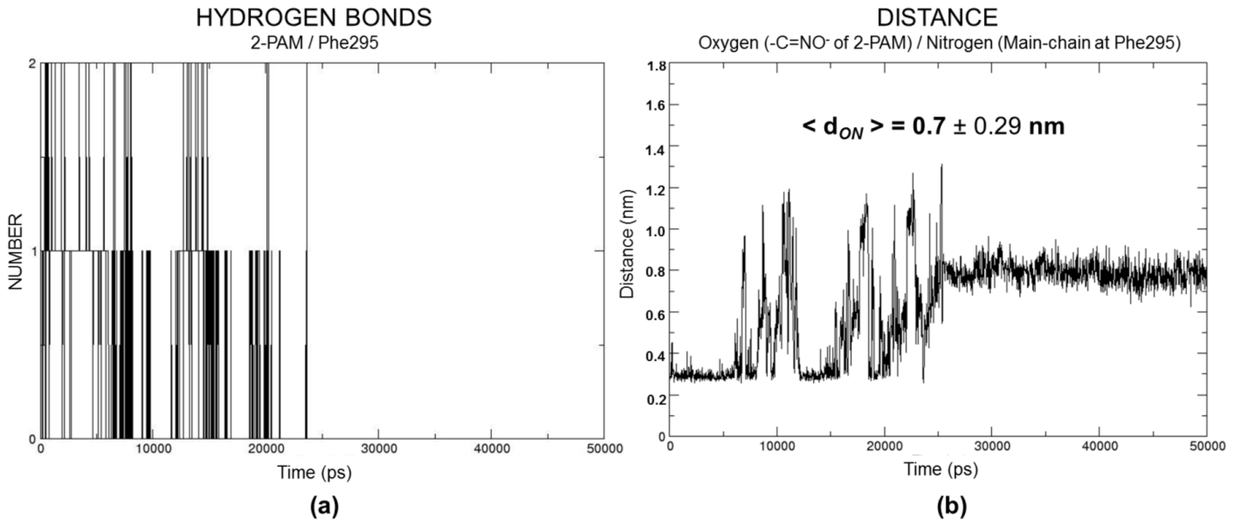

Figure S12. Plots of H-bonds between 2-PAM and atoms of Phe295 (a) throughout 50 ns of MD simulation. (b) Variation of distance *d* of the H-bond formed. "⟨ ⟩" represents the mean of *d* values.

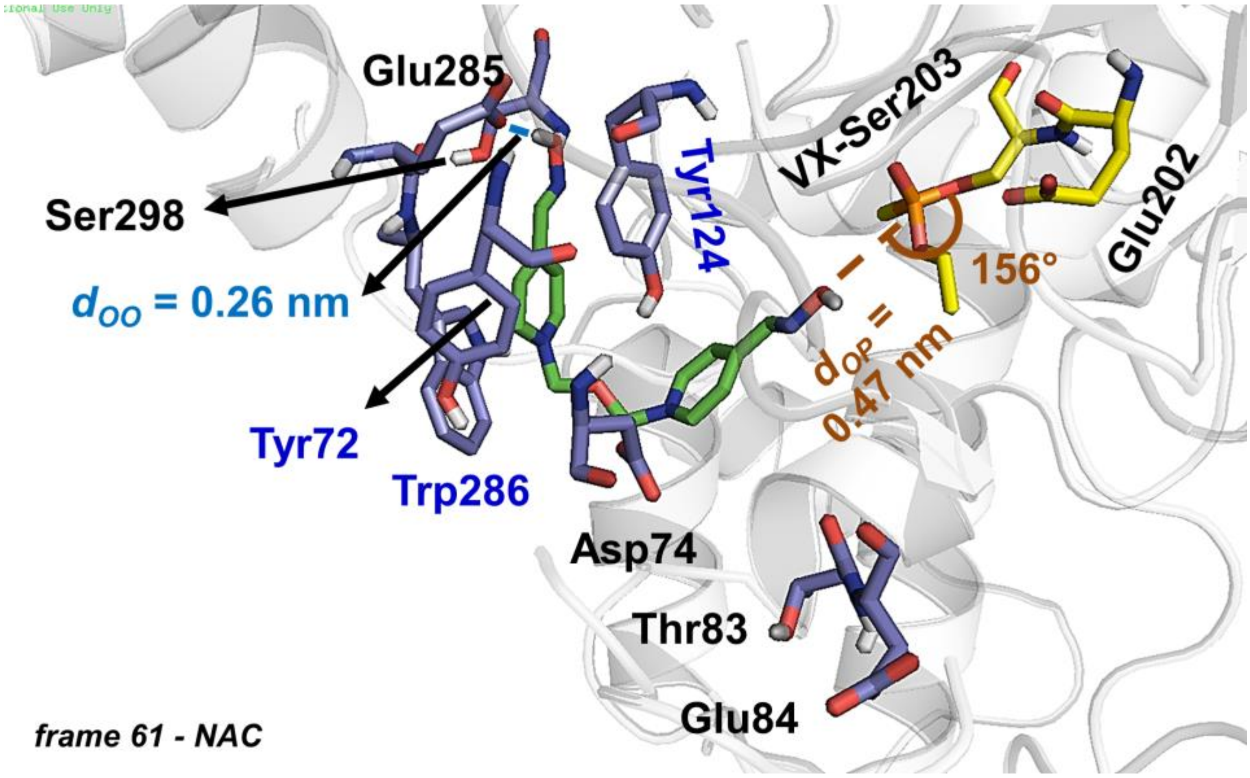

Figure S13. NAC frame of obidoxime protonated. Ligands are shown in green, residues of CAS in yellow and others in blue. H-bonds and $\boldsymbol{d_{OP}}$ are shown in blue and red dashed lines, respectively.

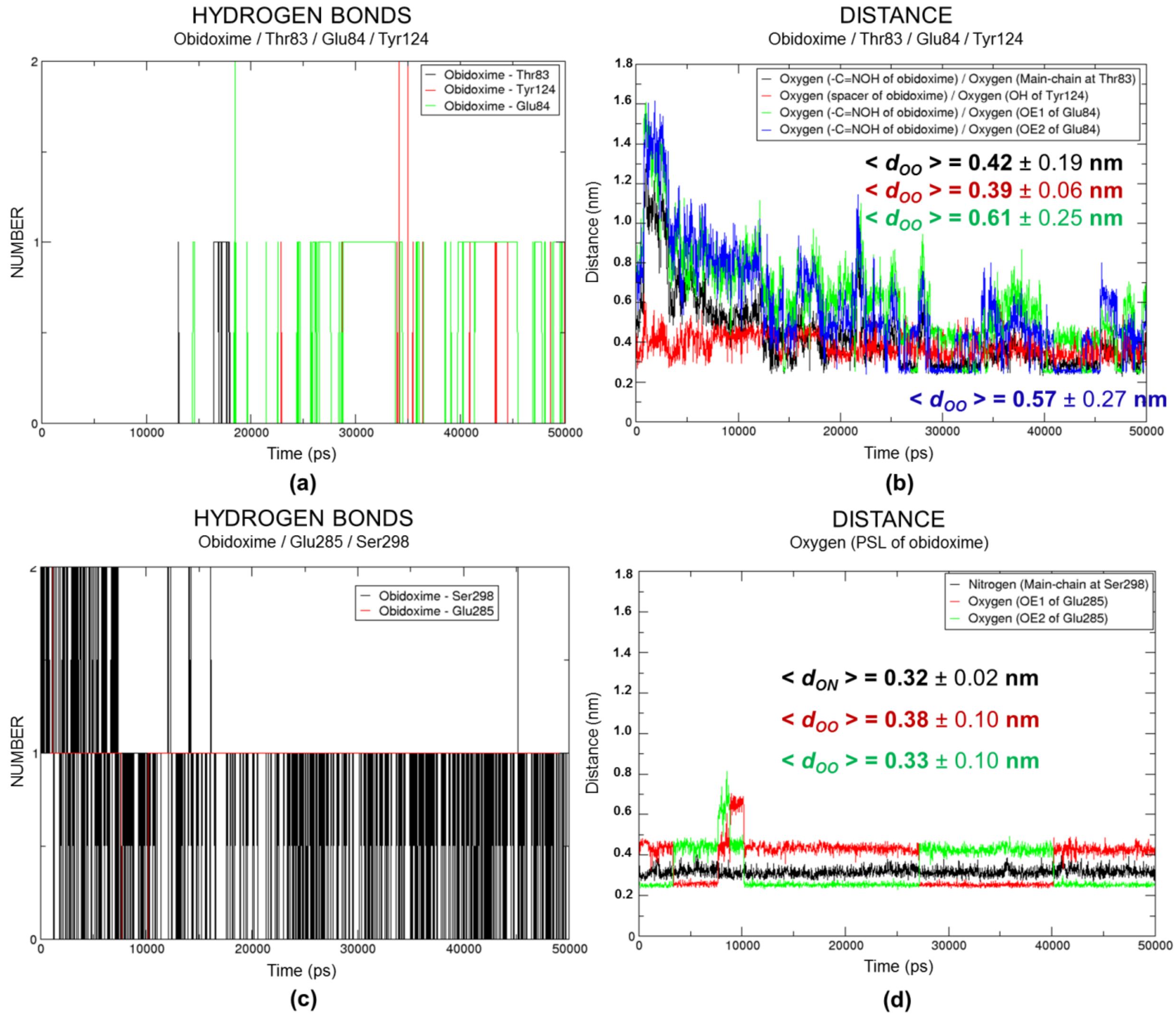

Figure S14. Plots of H-bond interactions formed amongst obidoxime protonated and residues Thr83, Glu84, Tyr124, Glu285, and Ser298 throughout 50 ns of MD simulation. In (a) and (b) H-bonds and *d* values formed amongst –C=NOH of obidoxime and the main-chain of Thr83 (in black), Glu84 (in green and blue) and Tyr124 (in red). In (c) and (d) H-bonds and *d* values formed amongst the PSL of obidoxime and the main-chains of Glu285 (in red and green) and Ser298 (in black). "⟨ ⟩" represents the average *d* values.

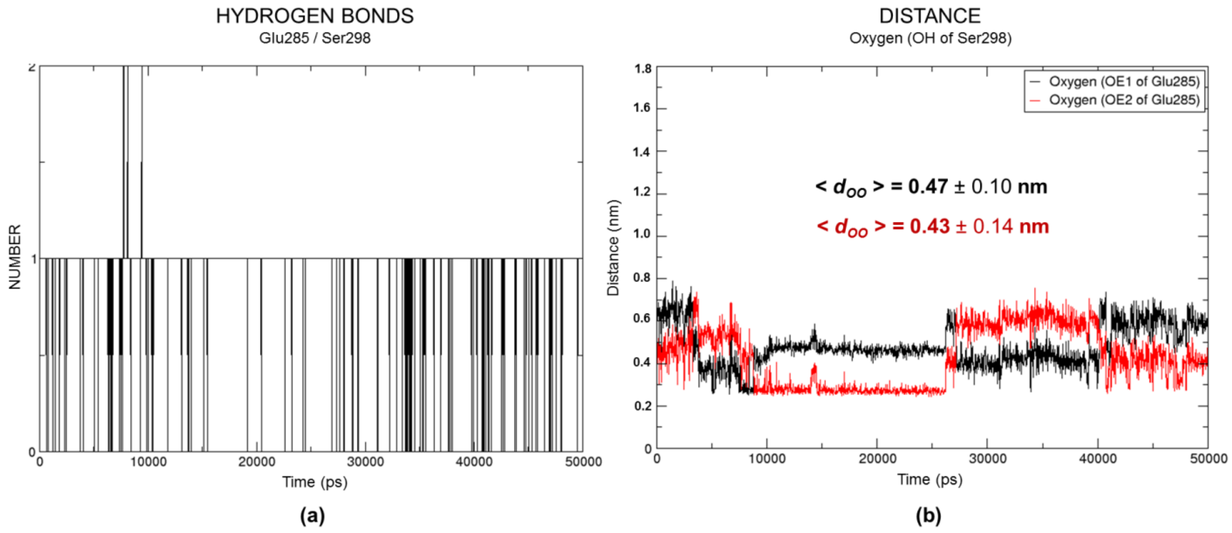

Figure S15. Plots of H-bond interactions formed between Glu285 and Ser298 throughout 50 ns of MD simulation of obidoxime. "⟨ ⟩" represents the average *d* values.

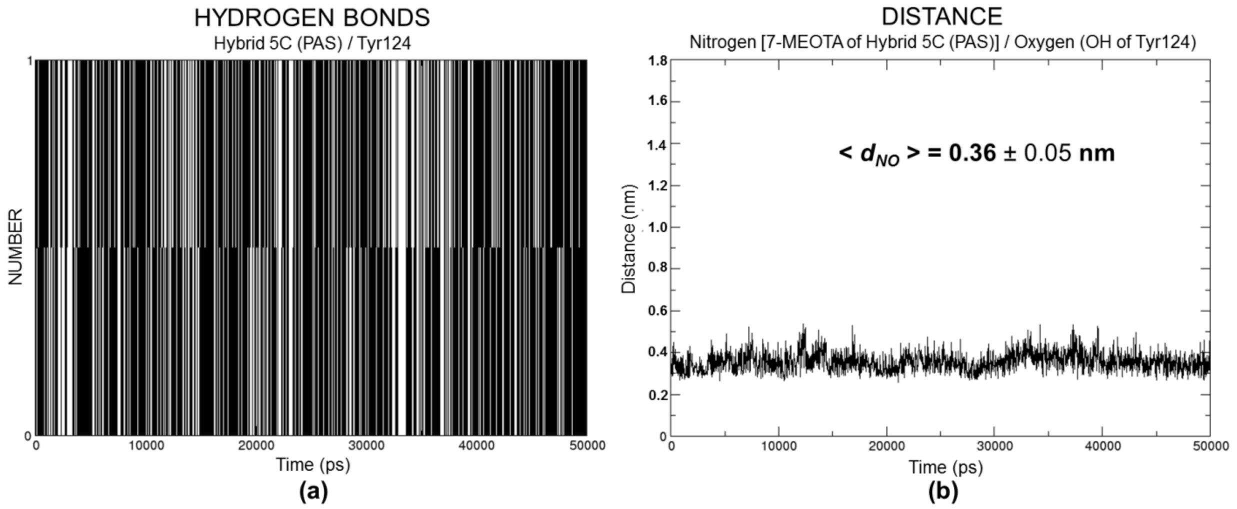

Figure S16. Plots of H-bond interactions between hybrid 5C and Tyr124 throughout 50 ns of MD simulation. "⟨ ⟩" represents the average *d* values.

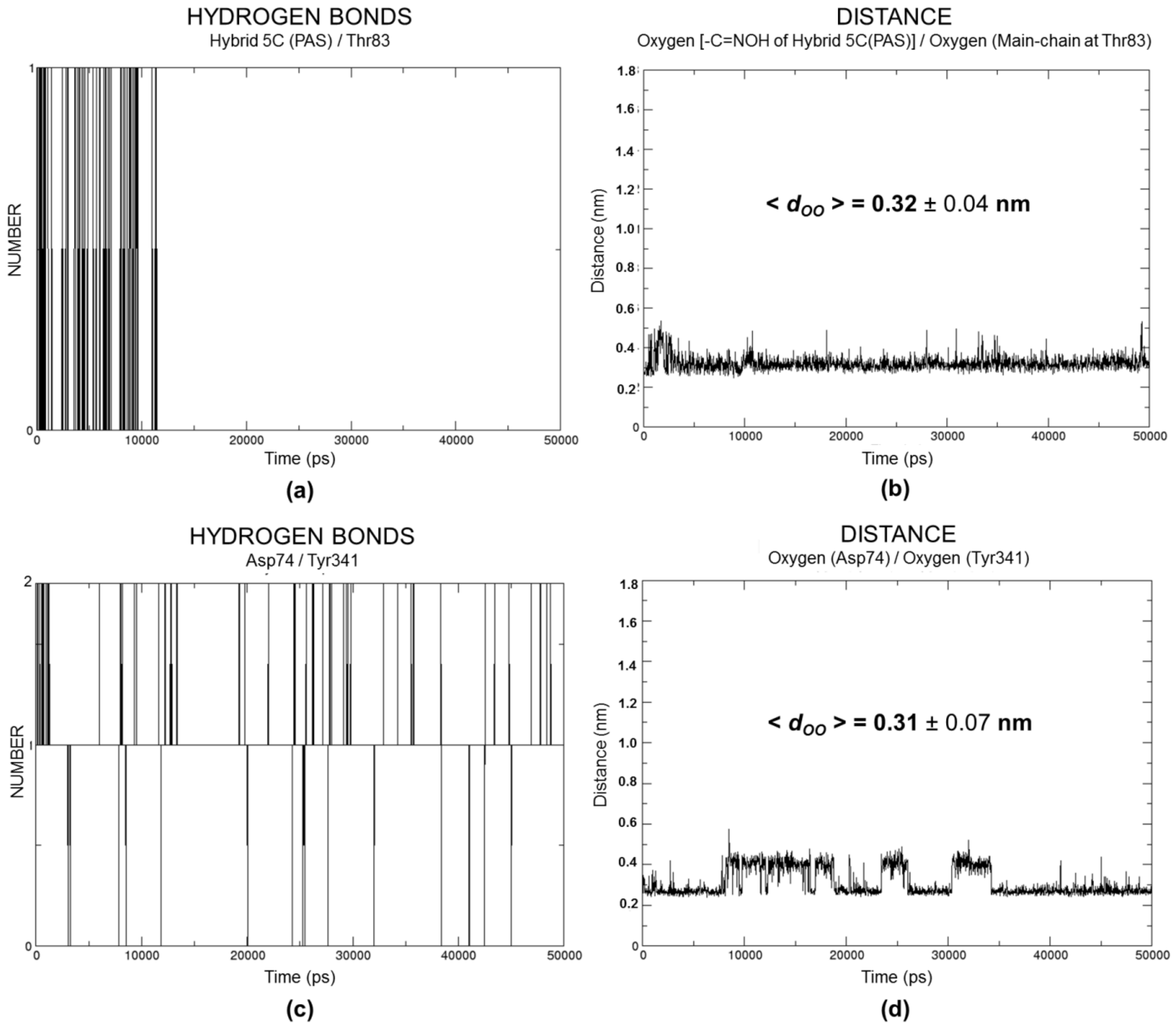

Figure S17. Plots of H-bond interactions formed amongst hybrid 5C, Asp74, Thr83, and Tyr341 throughout 50 ns of MD simulation. In (a) and (b) significant H-bonds formed in the first 10 ns and *d* values formed between the –C=NOH group and the main-chain of Thr83. In (c) and (d) H-bonds and *d* values formed between Asp74 and Tyr341. "⟨ ⟩" represents the average *d* values.

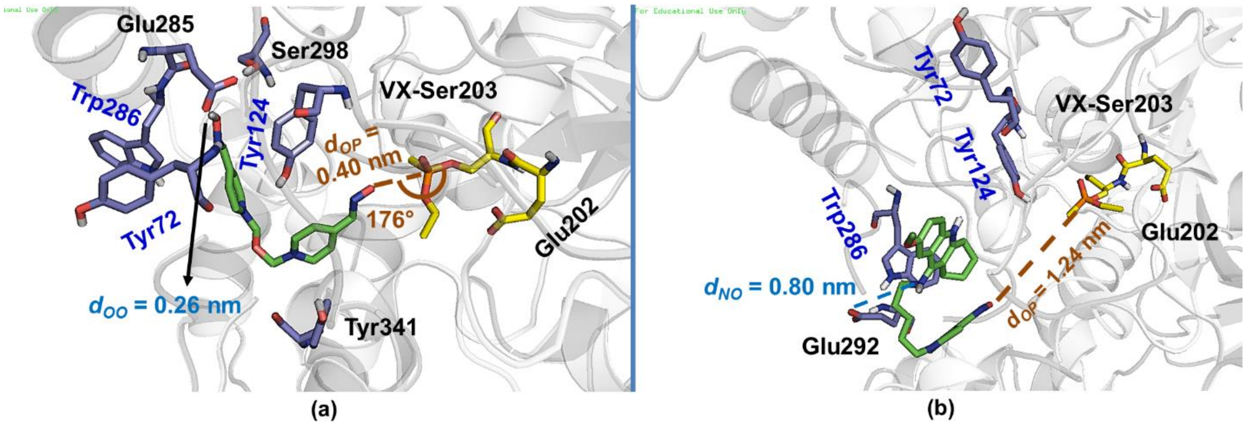

Figure S18. Comparison between the last frame of (a) obidoxime deprotonated and (b) hybrid 5C deprotonated. Ligands are shown in green, residues of CAS in yellow and others in blue. H-bonds and $\boldsymbol{d_{OP}}$ are shown in in blue and brown dashed lines, respectively.

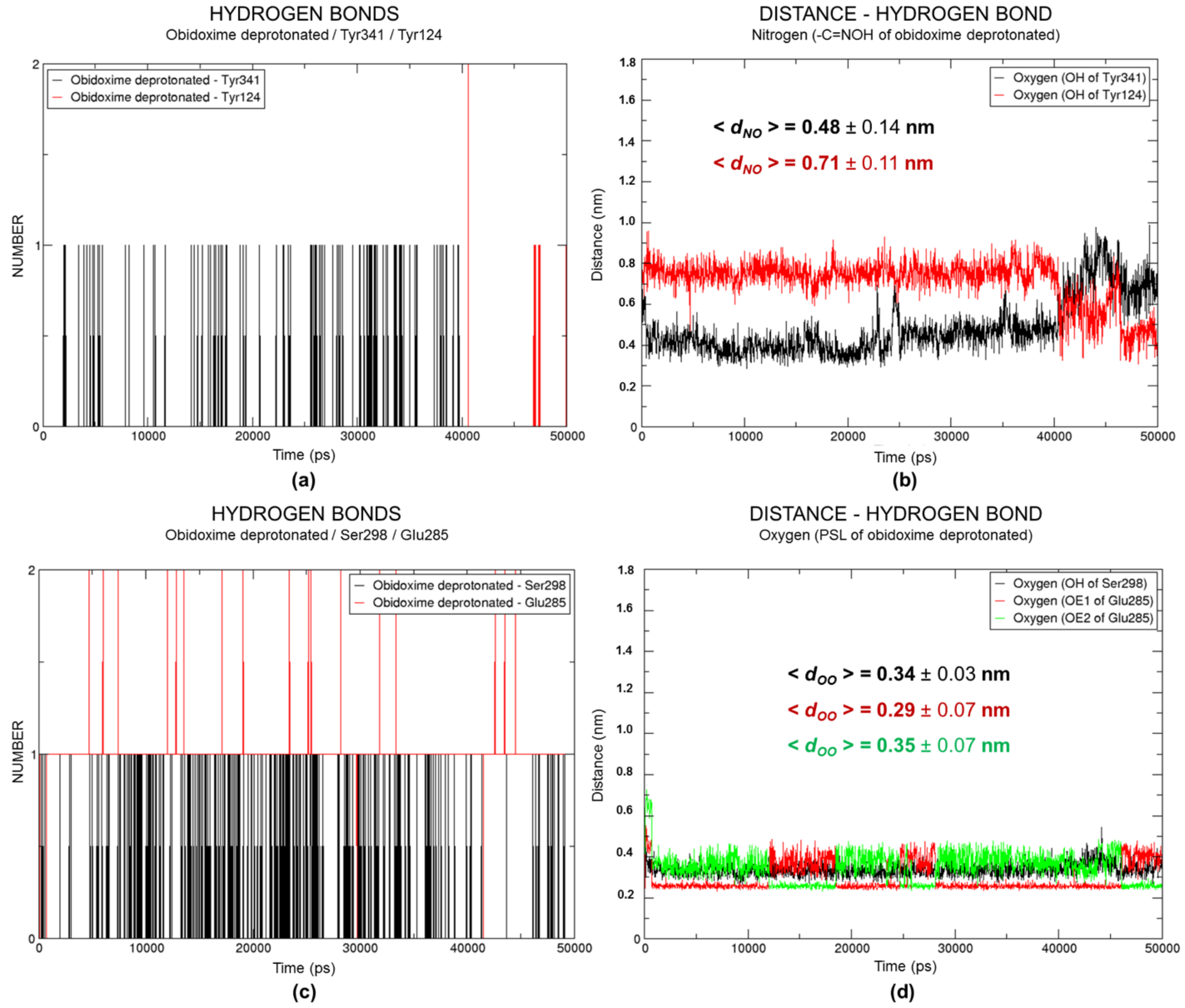

Figure S19. Plots of H-bond interactions formed amongst obidoxime deprotonated, Tyr124, Glu285, Ser298 and Tyr341throughout 50 ns of MD simulation. In (a) to (b) H-bonds and *d* values amongst the $-C=NO^-$ group of obidoxime and Tyr124 and Tyr341 are shown in black and red, respectively. In (c) and (d) H-bonds and *d* values formed amongst the PSL of obidoxime and Glu 285 and Ser298 are shown in red and green, respectively. "⟨ ⟩" represents the average *d* values.

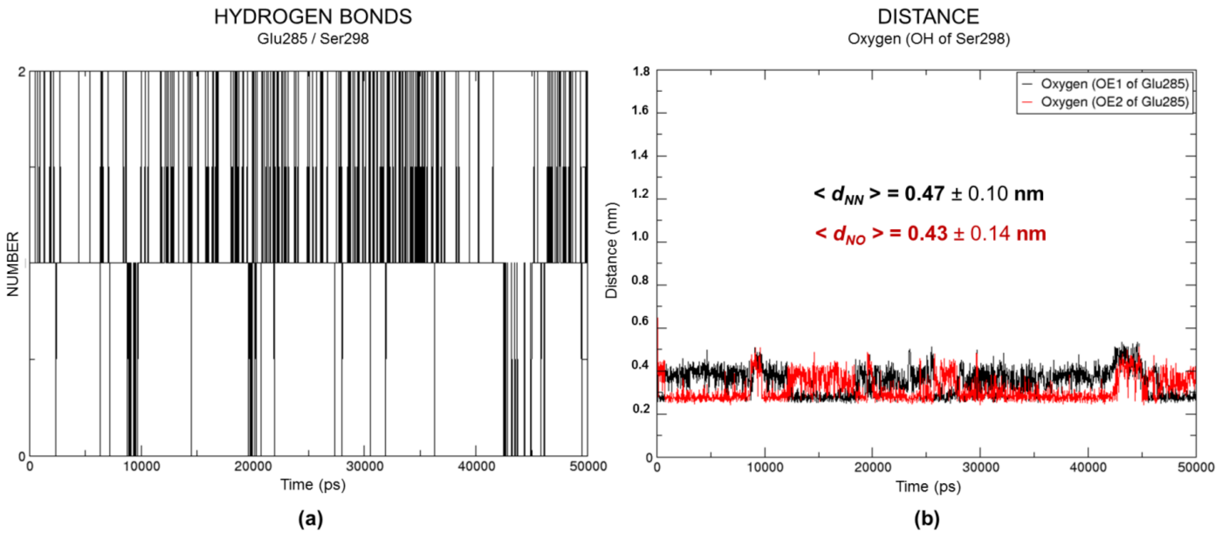

Figure S20. Plots of H-bond interactions formed between Glu285 and Ser298 throughout 50 ns of MD simulation of obidoxime deprotonated. "⟨ ⟩" represents the average *d* values.

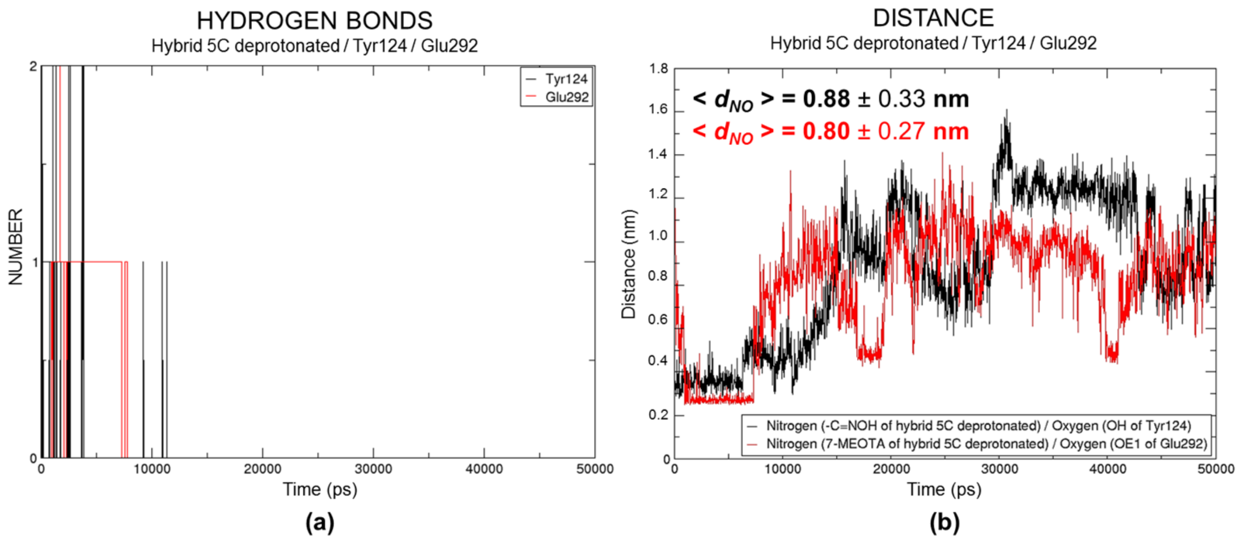

Figure S21. (a) Plots of H-bond interactions amongst hybrid 5C deprotonated and Tyr124 and Glu292 throughout 50 ns of MD simulation**.** H-bond formed (b) *d* values between the –C=NO^{-} group of the ligand and Tyr124, (in black) and between the PSL of ligand and Glu292 (in red). "⟨ ⟩" represents the average *d* values.

Table S1. The most significant contributions of key residues to $\langle \Delta H_{Binding} \rangle$ of ligands (Tables 1 and 2).

**Contributions per residue** [a, b, c]	**4-PA**	**2-PAM**	**Obidoxime**	**Hybrid 5C**
**Tyr72 (PAS** [g]**)**:				
$\langle \Delta E_{MM} \rangle$ [d]	- 3.35 ± 1.25	-	-	-
$\langle \Delta G_{Polar} \rangle$ [d]	0.67 ± 0.55	-	-	-
$\langle \Delta G_{Nonpolar} \rangle$ [d]	- 0.17 ± 0.12	-	-	-
$\langle \Delta \boldsymbol{H}_{\boldsymbol{Residue}} \rangle$ [e]	**- 2.86 ± 0.93**	-	-	-
**% of** $\langle \Delta \boldsymbol{H}_{\boldsymbol{Binding}} \rangle$ [f]	**4.58%**	-	-	-
**Asp74**:				
$\langle \Delta E_{MM} \rangle$ [d]	-	-	- 118.81 ± 21.30	- 101.99 ± 5.99
$\langle \Delta G_{Polar} \rangle$ [d]	-	-	77.60 ± 26.53	64.59 ± 13.13
$\langle \Delta G_{Nonpolar} \rangle$ [d]	-	-	- 0.97 ± 0.52	- 0.64 ± 0.22
$\langle \Delta \boldsymbol{H}_{\boldsymbol{Residue}} \rangle$ [e]	-	-	**- 42.18 ± 7.50**	**- 38.04 ± 9.72**
**% of** $\langle \Delta \boldsymbol{H}_{\boldsymbol{Binding}} \rangle$ [f]	-	-	**5.99%**	**5.29%**
**Glu84**:				
$\langle \Delta E_{MM} \rangle$ [d]	-	-	-72.34 ± 20.14	- 52.45 ± 4.72
$\langle \Delta G_{Polar} \rangle$ [d]	-	-	28 83 ± 17.96	13.31 ± 4.30
$\langle \Delta G_{Nonpolar} \rangle$ [d]	-	-	- 0.07 ± 0.08	- 0.02 ± 0.04
$\langle \Delta \boldsymbol{H}_{\boldsymbol{Residue}} \rangle$ [e]	-	-	**- 43.58 ± 4.45**	- 39.16 ± 1.73
**% of** $\langle \Delta \boldsymbol{H}_{\boldsymbol{Binding}} \rangle$ [f]	-	-	**6.19%**	5.44%
**Tyr124 (PAS** [g]**)**:				
$\langle \Delta E_{MM} \rangle$ [d]	- 8.31 ± 2.79	-	-	-
$\langle \Delta G_{Polar} \rangle$ [d]	5.03 ± 1.72	-	-	-
$\langle \Delta G_{Nonpolar} \rangle$ [d]	- 0.38 ± 0.19	-	-	-
$\langle \Delta \boldsymbol{H}_{\boldsymbol{Residue}} \rangle$ [e]	**- 3.65 ± 2.60**	-	-	-
**% of** $\langle \Delta \boldsymbol{H}_{\boldsymbol{Binding}} \rangle$ [f]	**5.85%**	-	-	-
**Glu202**:				
$\langle \Delta E_{MM} \rangle$ [d]	-	0.13 ± 1.72	-51.07 ± 2.98	-55.06 ± 1.71
$\langle \Delta G_{Polar} \rangle$ [d]	-	6.50 ± 5.36	4.46 ± 4.90	13.89 ± 3.12
$\langle \Delta G_{Nonpolar} \rangle$ [d]	-	- 0.01 ± 0.04	0.00	0.00
$\langle \Delta \boldsymbol{H}_{\boldsymbol{Residue}} \rangle$ [e]	-	**+ 6.61 ± 5.95**	**- 46.62 ± 5.95**	- 41.178 ± 3.200
**% of** $\langle \Delta \boldsymbol{H}_{\boldsymbol{Binding}} \rangle$ [f]	-	**11.32%**	**6.62%**	5.72%

[a] Only for energies within limits established in Figure 6.
[b] Calculated in kJ/mol.
[c] Values in bold for key contributions discussed in the text.
[d] Calculated through MM-PBSA methodology.
[e] Calculated as shown in equation 5.
[f] Calculated as shown in equation 4.
[g] Interactions within PAS as indicated in Figure 2.

Table S1. The most significant contributions of key residues to $\langle \Delta G_{Binding} \rangle$ of ligands (Tables 1 and 2) (**Cont.**).

**Contributions per residue** [a, b, c]	**4-PA**	**2-PAM**	**Obidoxime**	**Hybrid 5C**
**Glu285**:				
$\langle \Delta E_{MM} \rangle$ [d]	- 4.28 ± 2.18	-	-115.43 ± 12.69	-71.69 ± 3.75
$\langle \Delta G_{Polar} \rangle$ [d]	0.99 ± 6.50	-	63.28 ± 12.96	34.02 ± 7.93
$\langle \Delta G_{Nonpolar} \rangle$ [d]	- 0.04 ± 0.04	-	-0.06 ± 0.06	-0.07 ± 0.07
$\langle \Delta \boldsymbol{H}_{\boldsymbol{Residue}} \rangle$ [e]	**- 3.33 ± 6.35**	-	**- 52.21 ± 5.95**	-37.75 ± 6.43
**% of** $\langle \Delta \boldsymbol{H}_{\boldsymbol{Binding}} \rangle$ [f]	**5.34%**	-	**11.32%**	5.25%
**Trp286 (PAS** [g]**)**:				
$\langle \Delta E_{MM} \rangle$ [d]	- 8.31 ± 2.78	-	-	-
$\langle \Delta G_{Polar} \rangle$ [d]	2.76 ± 1.72	-	-	-
$\langle \Delta G_{Nonpolar} \rangle$ [d]	- 0.53 ± 0.20	-	-	-
$\langle \Delta \boldsymbol{H}_{\boldsymbol{Residue}} \rangle$ [e]	**- 6.346 ± 1.621**	-	-	-
**% of** $\langle \Delta \boldsymbol{H}_{\boldsymbol{Binding}} \rangle$ [f]	**9.73%**	-	-	-
**Ser298 (PAS** [g]**)**:				
$\langle \Delta E_{MM} \rangle$ [d]	- 8.75 ± 1.53	-	-	-
$\langle \Delta G_{Polar} \rangle$ [d]	5.16 ± 0.76	-	-	-
$\langle \Delta G_{Nonpolar} \rangle$ [d]	- 0.03 ± 0.04	-	-	-
$\langle \Delta \boldsymbol{H}_{\boldsymbol{Residue}} \rangle$ [e]	**- 3.62 ± 1.91**	-	-	-
**% of** $\langle \Delta \boldsymbol{H}_{\boldsymbol{Binding}} \rangle$ [f]	**5.80%**	-	-	-
**Phe338**:				
$\langle \Delta E_{MM} \rangle$ [d]	-	- 4.18 ± 2.06	-	-
$\langle \Delta G_{Polar} \rangle$ [d]	-	0.98 ± 0.90	-	-
$\langle \Delta G_{Nonpolar} \rangle$ [d]	-	- 0.17 ± 0.10	-	-
$\langle \Delta \boldsymbol{H}_{\boldsymbol{Residue}} \rangle$ [e]	-	**- 3.37 ± 1.40**	-	-
**% of** $\langle \Delta \boldsymbol{H}_{\boldsymbol{Binding}} \rangle$ [f]	-	**6.25%**	-	-
**Tyr341**:				
$\langle \Delta E_{MM} \rangle$ [d]	-	- 6.45 ± 2.00	-	-
$\langle \Delta G_{Polar} \rangle$ [d]	-	3.35 ± 1.12	-	-
$\langle \Delta G_{Nonpolar} \rangle$ [d]	-	- 0.55 ± 0.25	-	-
$\langle \Delta \boldsymbol{H}_{\boldsymbol{Residue}} \rangle$ [e]	-	**- 3.65 ± 6.25**	-	-
**% of** $\langle \Delta \boldsymbol{H}_{\boldsymbol{Binding}} \rangle$ [f]	-	**5.78%**	-	-

[a] Only for energies within limits established in Figure 6.
[b] Calculated in kJ/mol.
[c] Values in bold for key contributions discussed in the text.
[d] Calculated through MM-PBSA methodology.
[e] Calculated as shown in equation 5.
[f] Calculated as shown in equation 4.
[g] Interactions within PAS as indicated in Figure 2.

Table S2. The most significant contributions of key residues to $\langle \Delta G_{Binding} \rangle$ of obidoxime deprotonated and hybrid 5C deprotonated (Tables 1 and 2).

**Contributions per residue** [a, b, c]	**Obidoxime deprotonated**	**Hybrid 5C deprotonated**
**Asp74**:		
$\langle \Delta E_{MM} \rangle$ [d]	- 39.91 ± 6.81	- 29.04 ± 5.91
$\langle \Delta G_{Polar} \rangle$ [d]	15.58 ± 8.88	6.67 ± 8.23
$\langle \Delta G_{Apolar} \rangle$ [d]	- 0.20 ± 0.10	- 0.06 ± 0.13
$\langle \Delta \boldsymbol{G}_{\boldsymbol{Residue}} \rangle$ [e]	**- 24.52 ± 4.31**	- 22.44 ± 5.06
**% of** $\langle \Delta \boldsymbol{G}_{\boldsymbol{Binding}} \rangle$ [f]	**12.47%**	5.16%
**Glu285 (PAS** [g]**)**:		
$\langle \Delta E_{MM} \rangle$ [d]	- 68.23 ± 2.64	- 48.41 ± 12.27
$\langle \Delta G_{Polar} \rangle$ [d]	40.15 ± 3.99	26.32 ± 19.49
$\langle \Delta G_{Apolar} \rangle$ [d]	- 0.06 ± 0.07	- 0.33 ± 0.30
$\langle \Delta \boldsymbol{G}_{\boldsymbol{Residue}} \rangle$ [e]	**- 28.14 ± 4.78**	- 22.41 ± 9.72
**% of** $\langle \Delta \boldsymbol{G}_{\boldsymbol{Binding}} \rangle$ [f]	**14.30%**	5.16%
**Glu292**:		
$\langle \Delta E_{MM} \rangle$ [d]	- 37.16 ± 10.12	- 46.34 ± 18.60
$\langle \Delta G_{Polar} \rangle$ [d]	12.45 ± 13.48	16.93 ± 22.06
$\langle \Delta G_{Apolar} \rangle$ [d]	- 0.03 ± 0.11	-0.66 ± 0.50
$\langle \Delta \boldsymbol{G}_{\boldsymbol{Residue}} \rangle$ [e]	- 24.75 ± 4.82	**- 30.07 ± 5,07**
**% of** $\langle \Delta \boldsymbol{G}_{\boldsymbol{Binding}} \rangle$ [f]	12.58%	**6.92%**

[a] Only for energies within limits established in Figure 6.
[b] Calculated in kJ/mol.
[c] Values in bold for key contributions discussed in the text.
[d] Calculated through MM-PBSA methodology.
[e] Calculated as shown in equation 5.
[f] Calculated as shown in equation 4.
[g] Interactions within PAS as indicated in Figure 2.

Table S3. Binding energies computed for the NAC frames selected.

**LIGAND**	***Figure***	***Frame*** [a]	**t(ns)**	$\Delta H^{NAC\,frame}$ (kJ/mol) [b]	$\Delta H^{1^{st}frame}$ (kJ/mol) [b]	$\Delta\Delta H_{Binding}$ (kJ/mol) [c]
2-PAM	5 (a)	1764	35.28	- 69.108	- 37.830	- 31.278
Obidoxime deprotonated	5 (b)	2366	47.32	- 384.570	- 351.166	- 33.404
Hybrid 5C deprotonated	5 (c)	140	2.80	- 481.755	- 381.415	- 100.340
Obidoxime	S8	61	1.22	- 693.060	- 694.909	+ 1.845

[a] Selected with the highest ratio $\boldsymbol{\theta_{OPO}/d_{OP}}$.
[b] Calculated as equation 3 just for the respective frame.
[c] Calculated as equation 6.

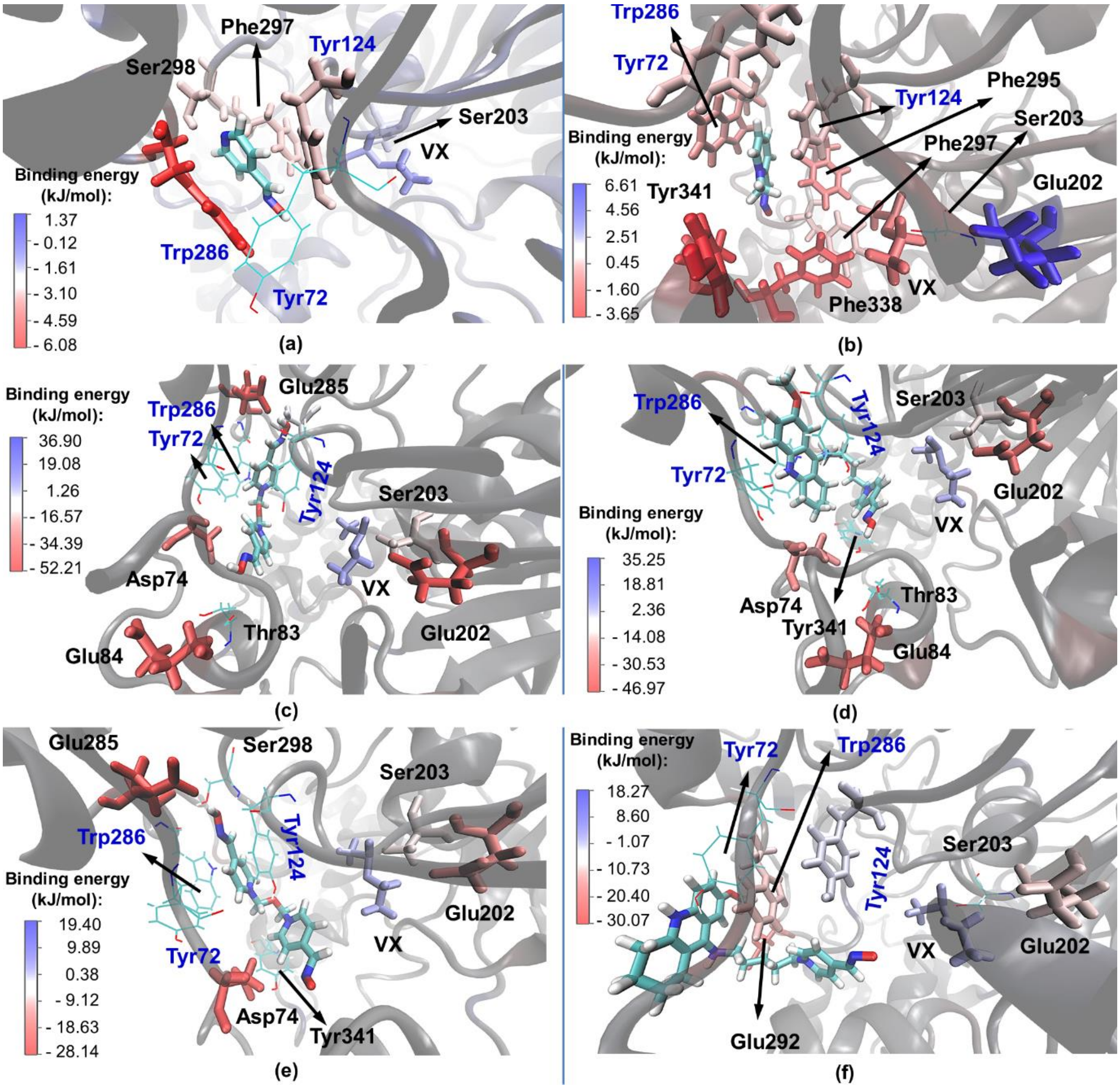

Figure S22. Comparisons amongst the most significant contributions $\langle \Delta H_{Residue} \rangle$ of key residues to $\langle \Delta H_{Binding} \rangle$, regarding (a) 4-PA, (b) 2-PAM, (c) obidoxime, d) hybrid 5C, (e) obidoxime deprotonated and (f) hybrid 5C deprotonated.
